# Cortical control of chandelier cells in neural codes

**DOI:** 10.3389/fncel.2022.992409

**Published:** 2022-10-10

**Authors:** Kanghoon Jung, Youngjin Choi, Hyung-Bae Kwon

**Affiliations:** Department of Neuroscience, Johns Hopkins University School of Medicine, Baltimore, MD, United States

**Keywords:** chandelier cells, axo-axonic cell, interneurons, cortical network, axon initial segment (AIS) inhibitory synapse, excitatory inhibitory balance, synaptic plasticity, schizhophrenia

## Abstract

Various cortical functions arise from the dynamic interplay of excitation and inhibition. GABAergic interneurons that mediate synaptic inhibition display significant diversity in cell morphology, electrophysiology, plasticity rule, and connectivity. These heterogeneous features are thought to underlie their functional diversity. Emerging attention on specific properties of the various interneuron types has emphasized the crucial role of cell-type specific inhibition in cortical neural processing. However, knowledge is still limited on how each interneuron type forms distinct neural circuits and regulates network activity in health and disease. To dissect interneuron heterogeneity at single cell-type precision, we focus on the chandelier cell (ChC), one of the most distinctive GABAergic interneuron types that exclusively innervate the axon initial segments (AIS) of excitatory pyramidal neurons. Here we review the current understanding of the structural and functional properties of ChCs and their implications in behavioral functions, network activity, and psychiatric disorders. These findings provide insights into the distinctive roles of various single-type interneurons in cortical neural coding and the pathophysiology of cortical dysfunction.

## Introduction

Various cortical processes depend on the dynamic interactions between excitation provided by glutamatergic pyramidal neurons (PyNs) and inhibition provided by interneurons (Hensch and Fagiolini, 2005). Interneurons releasing γ-aminobutyric acid (GABA) represent 10–20% of all cortical neurons in the brain (Rudy et al., 2011) and act as inhibitory nodes of neural circuits. The vast molecular diversity that exists among interneurons (Taniguchi, 2014) accounts for the variety of distinct cortical functions in the brain that collectively give rise to perception, cognition, and behavior. The chandelier cell (ChC) is a GABAergic interneuron cell type that has captured the interest of neuroscientists since its first discovery in the 1970s (Szentágothai and Arbib, 1974; Jones, 1975). The ChCs are also known as axo-axonic cells (Somogyi, 1977) due to their exclusive innervation of the axon initial segments of PyNs, a region for action potential generation.

Many discoveries have been made about the ChC's properties, functions, and implications in dysfunctional neural states, which indicate its vital role in proper cortical functioning. Distinct pathological states of ChCs are associated with neuropsychiatric disorders, such as schizophrenia (Rocco et al., 2017), epilepsy (DeFelipe, 1999), and autism spectrum disorder (Lunden et al., 2019). Although previous studies reported some controversies, such as whether the GABAergic neurotransmission of ChCs is inhibitory or excitatory (Szabadics et al., 2006; Woodruff et al., 2011) and whether its pathology in schizophrenia is contributory or compensatory (Rocco et al., 2017), recent advancements in genetic, optogenetic, and recording techniques have been applied to clarify these issues, illuminating the *in vivo* functions of ChCs in behaving animals and setting the stage for understanding its role in complex cognition.

The ChC is well-situated to mediate the balance between excitation and inhibition, given its innervation of the AIS that grants it effective, strategic inhibitory control over the excitatory activity of PyNs (Veres et al., 2014). The regulation of excitatory/inhibitory (E/I) balance by ChCs may have essential functions of preventing neuronal hyperexcitability and instantiating executive cognitive functions, as highlighted by respective pathophysiological states observed in epilepsy and schizophrenia. On one hand, dysfunctional ChCs are found in epileptic visual areas (Ribak, 1985), where seizures are generated due to unchecked propagation of excitatory activity. On the other hand, dysfunctional ChCs are found in the schizophrenic prefrontal cortex (Rocco et al., 2017), which is often associated with positive cognitive symptoms. Theoretical modeling has shown that the precise balance between inhibition and excitation in a neural network facilitates greater precision and efficiency in neural coding (Zhou and Yu, 2018). The failure of ChCs to mediate this function may underlie the disorganization of thought seen in schizophrenia.

In this review, we summarize the structural features of ChC morphology and connectivity, and neuroplasticity of axo-axonic synapses. We discuss functional features of ChC such as electrophysiological properties, synaptic effects, and neuromodulation of ChCs. We present recent discoveries about the ChC's *in vivo* functions in brain rhythms, behavioral states, and neural coding. Finally, we outline the potential pathophysiological mechanisms of ChCs in disrupted E/I balance and the corresponding implications in schizophrenia.

## Diversity of GABAergic interneuron types

GABAergic interneurons comprise 10–20% of all cortical neurons in the brain (Rudy et al., 2011) and have a fundamental role in operating neural circuitry by maintaining proper levels of excitability, synchronizing the firing of neuronal ensembles, controlling precise spike timing, and integrating synaptic inputs (Isaacson and Scanziani, 2011). These multifaceted functions can originate from interneuron heterogeneity in their morphology, connectivity, electrophysiology, and chemistry. We will review three major groups of GABAergic interneurons and the heterogeneity existing within each group, which spotlights ChCs as a single cell type within the taxonomy.

**Figure 1:**
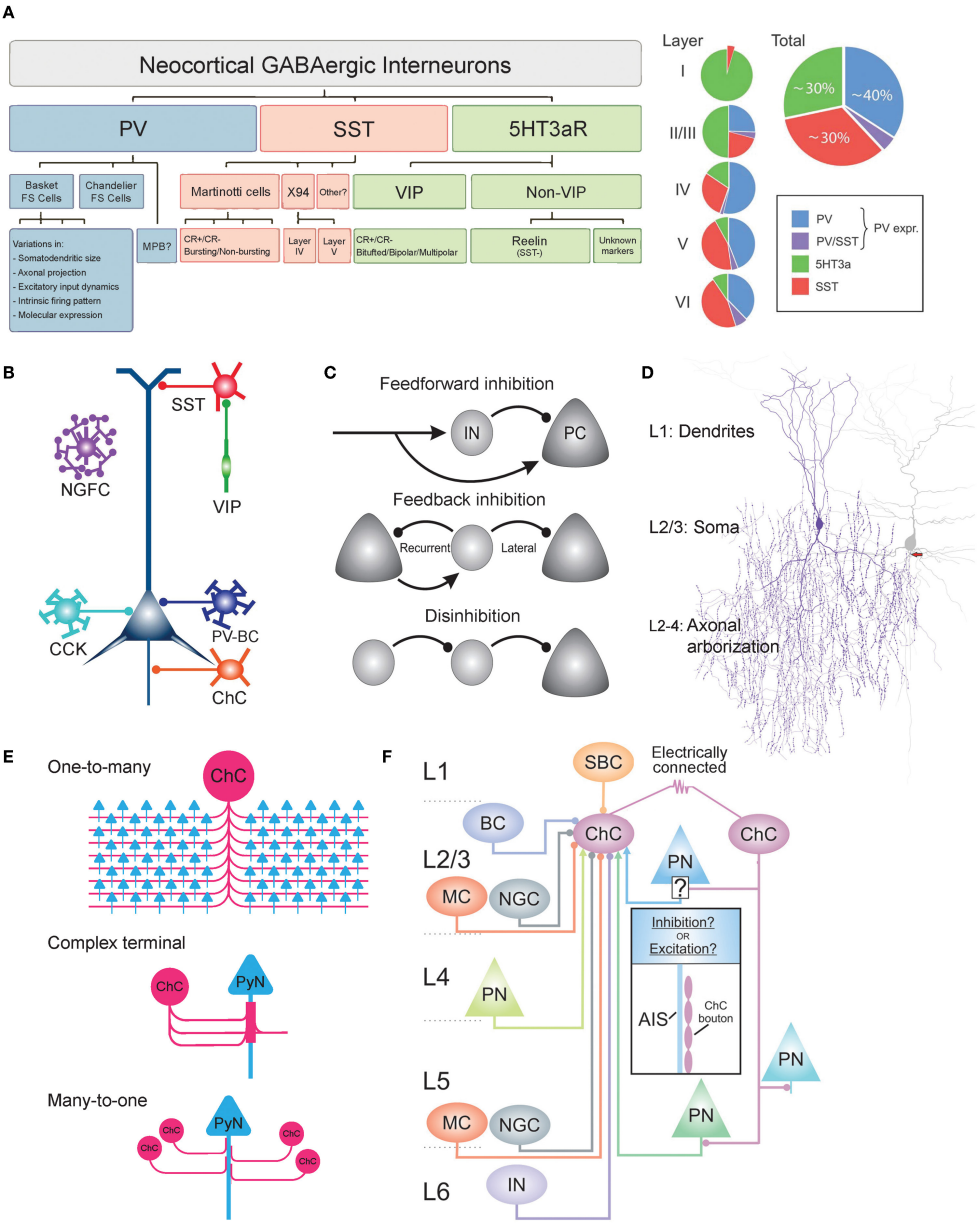
Heterogeneity of GABAergic interneurons and connectivity of ChCs. **(A)** Dendrogram and Venn diagram of neocortical GABAergic interneurons. Parvalbumin-expressing interneurons (PV-INs) account for ~40% of all GABAergic interneurons. PV-INs further divide into basket cells, chandelier cells, and multipolar bursting cells (MPBs). Somatostatin-expressing interneurons (SST-INs) account for ~30% of all GABAergic interneurons. The majority of SST-INs are Martinotti cells, which are morphological and electrophysiologically heterogeneous and can be further classified as calretinin-positive (CR+) or calretinin-negative (CR–). 5HT3aR-expressing interneurons account for ~30% of all GABAergic interneurons and are further divided into vasoactive intestinal peptide (VIP)-positive or VIP-negative. VIP-positive neurons display heterogeneity in their morphology and firing patterns. The majority of VIP-negative neurons express reelin. Adapted from Rudy et al. (2011). **(B)** Schematic of subcellular innervations of various GABAergic interneuron types on pyramidal neurons (PyNs). PV-expressing basket cell (PV-BC) innervates the dendrite and soma of PyNs. ChC exclusively innervates the axon initial segment of PyNs. SST-INs target the dendrites of PyNs. VIP-positive neurons commonly innervate SST-INs to disinhibit PyNs. Adapted from Taniguchi (2014). **(C)** Common circuit motifs utilized by GABAergic interneurons. In feedforward inhibition, an external source makes excitatory synapses onto both local PyNs and the GABAergic interneurons. FFI is used by ChCs and other GABAergic interneurons. Feedback inhibition occurs when GABAergic interneurons exert inhibition on local PyNs that initially provided excitation (recurrent) or other neighboring neurons that did not participate in the recruitment of the GABAergic interneuron (lateral). In disinhibition, the principal target of an interneuron is another interneuron, suppressing its inhibitory activity onto PyNs. VIP neurons inhibit SST-INs to disinhibit PyNs. Adapted from Tremblay et al. (2016). **(D)** Dendritic, somatic, and axonal morphology of neocortical ChCs. Dendrites radially arborize within a diameter of 100–150 μm, consisting of two main trunks: upper (branching to L1) and lower (branching to L4). The main axon descends 50–100 μm before profusely branching within a diameter of 100–200 μm, occupying L2–L4. The axon terminal segments consist of vertically oriented multiple boutons, which are each 1–2 μm in size and exclusively innervate the axon initial segment (AIS) of PyNs. The soma is oval in shape, found mostly in upper L2/3. Adapted from Wang et al. (2016). **(E)** Output connectivity features of ChCs. One-to-many connectivity to PyNs (top), in which a single ChC innervates many PyNs (35–50% of all PyNs that reside within the ChC's axonal field). Complex axon terminals (middle), in which multiple ChC cartridges converge onto the same region of PyN AIS to form a cylindrical-shaped axon terminal. Many-to-one connectivity to PyNs (bottom), in which multiple ChCs innervate the same PyN AIS. **(F)** Schematic of laminar distribution of inputs to cortical L2/3 ChCs. Circular and arrow tips indicate inhibitory and excitatory inputs, respectively. ChCs may be innervated by single-bouquet cells (SBC) from L1; Martinotti cells (MC), neurogliaform cells (NGC), other ChCs *via* gap junctions, basket cells (BC), and pyramidal neurons (PN) from L2/3; PN from L4; NGC and PN from L5; Unknown interneuron type from L6. Adapted from Wang et al. (2021).

First, parvalbumin-expressing interneurons (PV-INs) account for ~40% of GABAergic interneurons (Tamamaki et al., 2003; Fogarty et al., 2007; Figure 1A). PV-INs consist mostly of basket cells (PV-BCs) that exert perisomatic inhibition targeting the soma and proximal dendrites of PyNs (Martin et al., 1983; Kawaguchi and Kubota, 1997; Figure 1B). PV-BCs share common excitatory inputs with their target PyNs that they innervate, demonstrating feedforward inhibition (Willems et al., 2018; Figure 1C; top). In addition, PV-INs neurons can be reciprocally connected with PyNs to provide feedback inhibition (Grosser et al., 2021; Figure 1C; middle). In addition to PV-BCs, cholecystokinin-expressing BCs (CCK-BCs) constitute a smaller proportion of GABAergic interneurons. Both types of BCs innervate perisomatic domains with similar GABA-A receptor subunit composition contents (Kerti-Szigeti and Nusser, 2016) and mediate similar potencies of perisomatic inhibition to control PyN firing (Andrási et al., 2017). However, these two BC cell types receive excitatory inputs from PyNs with distinct properties (Andrási et al., 2017), and CCK-BCs display slower firing rates (~30 Hz) than PV-BCs (~110 Hz) (Szabó et al., 2010; Barsy et al., 2017). ChCs have been traditionally considered to be PV-INs, despite evidence of little to no expression of PV (Taniguchi et al., 2013). ChCs are distinct from BCs in their exclusive connectivity to the axon initial segment (AIS) of PyNs (Somogyi, 1977; DeFelipe et al., 1985; Figure 1B). Electrophysiologically, PV-INs are fast-spiking, exhibiting high-frequency action potentials and little adaptation (Xu and Callaway, 2009). Yet, multipolar bursting (MPB) neurons, PV-INs found in the upper L2, do not display the characteristic fast-spiking firing pattern of other PV-INs (Blatow et al., 2003).

Second, somatostatin-expressing INs (SST-INs) comprise ~30% of GABAergic interneurons (Lee et al., 2010; Figure 1A). SST-INs consist mostly of Martinotti cells (MCs) that exert dendritic inhibition targeting the distal apical dendrites of post-synaptic neurons (Karube et al., 2004; Figure 1B). Neocortical MCs project axons that horizontally bifurcate in L1 (Wang et al., 2004). MCs can be subdivided into two types, based on their expression of the calcium-binding protein calretinin (CR). CR+ and CR- MCs have been shown to exhibit differences in their dendritic morphology as well as input connectivity (Xu et al., 2006; Kapfer et al., 2007). The electrophysiology of these neurons is characterized as regular-spiking with adaptation or burst-spiking (Kawaguchi and Kubota, 1997). X94 cells are another type of SST-INs, distinct from MCs in their anatomy and electrophysiology. They are found in L4,5 and innervate L4, and they display lower input resistance along with shorter duration spikes and a stuttering firing pattern (Ma et al., 2006). In addition to the MCs and X94 cells, there are likely more subtypes of SST-INs, given observations of varying firing patterns, molecular markers, and connectivity (Xu et al., 2006; Gonchar et al., 2008).

Third, ionotropic serotonin-receptor-expressing INs (5HT3aR-INs) constitute ~30% of INs (Lee et al., 2010; Figure 1A). 5HT3aR-INs are divided into vasoactive intestinal peptide (VIP)-expressing INs (40% of 5HT3aR-INs) and non-VIP-expressing INs (~60% of 5HT3aR-INs) (Lee et al., 2010). Both are diverse among themselves. The majority of VIP+ INs preferentially innervate SST-INs and in turn disinhibit PyNs through the inhibition of SST-INs (Acsády et al., 1996; Pfeffer et al., 2013; Figure 1C; bottom). VIP+ INs are further classified by their morphology (bitufted, bipolar, or multipolar) (Miyoshi et al., 2010) and coexpression of calretinin (CR) (Cauli et al., 2000), which overlaps with molecular profiles of SST-IN subtypes mentioned above. VIP- INs are further classified by their expression of reelin (e.g., neurogliaform cells, ~80% of VIP- neurons), which is also coexpressed in some SST-IN subtypes (Lee et al., 2010).

Here we have outlined the substantial heterogeneity and overlap that exist among the three major interneuron groups: PV, SST, and 5HT3aR. Such heterogeneity poses a challenge in defining single cell types, which is a prerequisite to dissecting distinct synaptic properties and functions in a cell-type-specific manner. To overcome the challenge, there has been an increasing effort to identify and classify homogeneous interneuron types. As an example of this ongoing endeavor, we spotlight the development of the various methods used to identify the ChC. In early studies of ChCs (DeFelipe et al., 1985), Golgi staining was used to visualize the fine anatomical details of the cell, which revealed its chandelier-like axonal arborization geometry ChC morphology, which allowed researchers to distinguish it from other interneuron types. Later immunohistochemical techniques revealed the immunoreactivity of ChCs to various proteins, such as GAT-1, PV, calbindin, corticotropin-releasing factor, and ankyrin-G (Howard et al., 2005). Their immunoreactivity to PV was observed in various brain regions, including the visual cortex (Lewis and Lund, 1990), sensory-motor cortex (DeFelipe et al., 1985), prefrontal cortex (Taniguchi et al., 2013), entorhinal cortex (Schmidt et al., 1993), and hippocampus (Sik et al., 1993). These widespread observations led researchers to regard ChCs as a subset of PV-expressing interneurons (Rudy et al., 2011). However, a later study showed that only ~50 and ~15% of ChCs in the somatosensory cortex and the medial prefrontal cortex (mPFC), respectively, were immunoreactive for PV (Taniguchi et al., 2013), demonstrating a significant heterogeneity of PV-expression among ChCs. In addition, other molecular markers of ChCs such as CRF (Lewis and Lund, 1990) and calbindin (Rio and DeFelipe, 1997) have demonstrated considerable heterogeneity among ChCs, together posing challenges in determining a homogeneous set of molecular features of the cell.

In recent years, advancements in genetics and transcriptomics have enabled a more rigorous investigation of the molecular profile of single cell types and granted genetic access to ChCs, giving way to significant discoveries about their function in synaptic inhibition and cortical processing. Using single-cell RNA sequencing (scRNA-seq) and cluster analysis, the transcriptional profile of ChCs was systematically sequenced and transcriptional differences from other GABAergic interneurons were revealed. For example, cortical ChCs showed their high expression levels of several genes, including cell adhesion molecule UNC5b, γ-subunit of GABA-A receptors, calcium-binding protein Rasgrp1, and cGMP-dependent PKG Prkg (Paul et al., 2017). In addition, hippocampal ChCs were identified by their high expression levels of unique marker genes Ntf3 and Sntb1 (Yao et al., 2021). Furthermore, a multimodal cell census and atlas of the mammalian primary motor cortex have shown that ChCs indeed constitute a highly distinct neuronal cell type based on their transcription profiles (BICCN, 2021). Lastly, the PV-Vipr2 cell type identified by scRNA-seq data analysis was shown to correspond to the phenotypic ChC cell type, and the genetic marker Vipr2 was used to develop the transgenic mouse line Vipr2-IRES2-cre, allowing direct genetic access to ChCs (Tasic et al., 2018). The recent progress has strengthened the long-standing understanding of the ChC as a distinct single cell type with genetic profile in detail and increased accessibility to ChCs to study its functions *in vivo* in transgenic ChC specific mice. The study of ChCs thus marks a progress in detangling the complexity of GABAergic INs and serves as a platform to investigate the specific role of single IN types in brain function.

## Morphology and connectivity

ChCs are identified by their unique chandelier-like axonal structures which are preserved across various species such as cats, rodents, and monkeys (Somogyi et al., 1982). ChCs are found in various neocortical regions including the dorsolateral prefrontal cortex (DLPFC) (Schoonover et al., 2020), motor cortex (Somogyi et al., 1982), visual cortex (Somogyi et al., 1982), and somatosensory cortex (Zhu et al., 2004). In the neocortex, ChCs are most abundant in cortical layer 2/3 (L2/3) (Somogyi, 1977) where ChCs account for 2% of all GABAergic interneurons (Jiang et al., 2015). ChCs are also found in subcortical regions, such as the hippocampus—CA3 (Gulyás et al., 1993), CA1 (Somogyi et al., 1983), and dentate gyrus (Soriano and Frotscher, 1989)—and the basolateral amygdala (BLA) (McDonald, 1982).

### Morphology

The soma of ChCs is small and fusiform-shaped, with roughly a width of 8–10 μm and length of 16–20 μm (Somogyi et al., 1982; Figure 1D). ChC dendrites branch radially from the soma within a cylinder of 100–150 μm diameter (Somogyi et al., 1982). Two main dendritic trunks originate from the upper and lower regions of the soma, giving the ChC a bitufted morphology. In the neocortex, the upper main trunk ascends to L1 (Xu and Callaway, 2009) and the lower descends to L4 (Somogyi, 1977). On the dendritic shaft, a moderate number of drumstick-shaped spines can be found (Somogyi, 1977). The main ChC axon originates from the lower main dendritic trunk (Somogyi et al., 1982) or from the soma base (Lewis and Lund, 1990) and descends 50–100 μm before arborizing profusely and expansively, with an axonal field that covers L2/3/4 within a cylinder of 100–200 μm width (Somogyi et al., 1982). The main axons are myelinated (Somogyi et al., 1982). The axonal arbor of ChC axons consists of as many as 400 rows of horizontal collaterals (DeFelipe et al., 1985) that terminate in vertically oriented axon terminals also called “cartridges” (Szentágothai, 1975; Somogyi, 1977), which contain multiple synaptic boutons, ranging from 2~12 in the mouse neocortex (Inan et al., 2013) and ~8.4 in the mouse basolateral amygdala (Veres et al., 2014). The length of cartridges ranges between 10 and 50 μm, and the average synaptic bouton is 1–2 μm in size (Somogyi, 1977). These distinctive features of the ChC's axon, which resembles a chandelier, provide the basis for its name. Despite these morphological features that are largely uniform among ChCs across various brain areas and species, it is noteworthy that a recent study using high-resolution, large-volume light microscopy revealed that there are morphologically distinct subtypes based on variations in its dendritic and axonal morphology and laminar arrangement among ChCs (Wang et al., 2019). The laminar position and local geometry of dendrites and axons could determine the recruitment of different ChCs by input streams and the innervation of different PyN populations by ChCs, respectively (Wang et al., 2019). Therefore, the fine granularity of the ChC morphology and laminar distribution may indicate a potential functional heterogeneity among the ChC cell type.

### Output connectivity

The most distinctive feature of ChCs is their exclusive innervation of PyNs at the axon initial segment (AIS) (Somogyi, 1977; DeFelipe et al., 1985), where action potential is generated (Stuart and Sakmann, 1994; Ogawa and Rasband, 2008). ChC cartridges vertically align themselves along the AIS of PyNs, forming multiple synaptic connections through numerous axonal boutons per AIS. The distribution of ChCs synapses is not uniform along the AIS, as they have been shown to preferentially contact a particular portion of AIS with a cross-sectional diameter of 0.5–1 μm (Vereczki et al., 2016) and distance of 20–40 μm from the PyN soma (Veres et al., 2014), which has been shown to exhibit the lowest threshold for action potential generation (Veres et al., 2014). ChC cartridges display a characteristic tendency to climb upwards along the length of the PyN AIS, with proximal boutons targeting the distal ends of the AIS (Fairén and Valverde, 1980; Peters et al., 1982; Howard et al., 2005). In these ways, ChCs cartridges strategically organize their synapses along the PyN AIS to maximize their inhibitory control. The strength of inhibition by ChCs onto PyNs has been shown to be correlated to the number of boutons contacting the AIS, with greater numbers giving rise to more effective inhibition of PyN activity (Veres et al., 2014). Specifically, 10–12 ChC synapses onto the AIS are sufficient to reduce the firing probability of PyNs by 95% and thereby veto the generation of action potentials (Veres et al., 2014).

A single ChC densely innervates hundreds of PyNs (Figure 1E; top), specifically 35–50% of all PyNs that reside within its axonal field (Inan et al., 2013). This number ranges between 50 and 200 PyNs in the neocortex (Somogyi et al., 1982), 600–650 PyNs in the basolateral amygdala (Vereczki et al., 2016), and up to 1,200 PyNs in the hippocampus (Li et al., 1992). In some brain regions, multiple cartridges of a single ChC converge onto the same PyN AIS to create a complex cylindrical axon terminal (Fairén and Valverde, 1980; Inda et al., 2009; Figure 1E; middle). Furthermore, multiple ChCs can innervate a single PyN AIS, ranging from ~4 ChCs in the mouse somatosensory cortex (Inan et al., 2013), ~13 In the mouse visual cortex (Schneider-Mizell et al., 2021), and 6–7 in the mouse basolateral amygdala (Vereczki et al., 2016; Figure 1E; bottom). The convergent connections of ChCs to PyNs arise during post-natal development when the AIS is innervated by an excessive number of AIS-preferring axons of multiple ChCs, which show target preference by post-natal day 14 (P14), as well by non-AIS-preferring axons which are eliminated by P28 (Gour et al., 2021). These meticulous connectivity patterns allow ChCs to precisely and powerfully control the activities of PyN populations (Veres et al., 2014).

### Input connectivity

ChCs receive excitatory and inhibitory inputs from a variety of cortical layers through synapses located primarily on the dendritic spines, less commonly on the soma, and none on the AIS (Somogyi et al., 1982). Specifically on their dendrites, ChCs receive a similar density yet fewer number of excitatory glutamatergic inputs by PyNs when compared to BCs, due to the less elaborate branching of the ChC's dendritic trees (Papp et al., 2013). As a result, ChCs display a rate of spontaneous excitatory post-synaptic current that is lower than that of BCs (Papp et al., 2013).

Various brain regions have been studied to determine the distribution of local inputs to L2/3 ChCs (Figure 1F). In the primary visual cortex (V1) of adult mice, octuple whole-cell recordings revealed that ChCs receive monosynaptic inhibitory input from single-bouquet cells (SBCs) in L1, BCs in L1, and MCs in L1 and L5 (Jiang et al., 2015). In the prelimbic cortex of adult mice, the laminar distribution of monosynaptic excitatory input to ChCs by PyNs was studied *via* optogenetic stimulation of PyNs in various layers. L2/3 ChCs receive the greatest excitatory input from more distant layers, namely L3 and L5, and significantly less input from L1 (Lu et al., 2017). Global connectivity to prelimbic ChCs was examined using trans-synaptic rabies tracing. At a network level, prelimbic ChCs receive monosynaptic excitatory input from the contralateral prelimbic cortex and mediodorsal, anteromedial, and ventromedial thalamic nuclei, and cholinergic input from the diagonal band of the basal forebrain (Lu et al., 2017). Furthermore, prelimbic ChCs were shown to receive the strongest inhibitory input from L1 (Lu et al., 2017). In the primary somatosensory cortex (S1) of adult mice, laser scanning photostimulation was used to determine the distribution of input strengths from various layers. S1 ChCs receive the strongest excitatory input from local L2/3 and L5 PyNs; no significant excitatory input from L4 PyNs; the strongest inhibitory input from L1 followed by L2/3; no significant inhibitory input from L4 and L5A; and weak inhibitory input from L5B and L6 (Xu and Callaway, 2009).

Input to ChCs is not limited to chemical synapses. Neighboring L2/3 ChCs have been shown to be electrically coupled through gap junctions which may facilitate their concerted activity (Woodruff et al., 2011; Figure 1F). Less commonly, ChCs are connected by gap junctions with adjacent BCs (Woodruff et al., 2011). In addition, in the mPFC, the firing of ChCs induced glutamatergic excitation that activated a nearby ChCs and back to itself, indicating that nearby ChCs may di-synaptically activate one another *via* an intermediate PyN (Taniguchi et al., 2013). The electrical coupling and di-synaptic excitation among local ChCs may have a role in promoting the synchronized inhibition of PyN populations, a property that has been observed in other GABAergic interneurons (Beierlein et al., 2000). Additionally, electrical coupling among ChCs *via* gap junctions may instead have a more complex role of desynchronizing the firing of ChC populations, a function that was recently shown to be possible when GABAergic interneurons coupled only *via* gap junctions evoke large, slow, inhibitory gap junction potentials with high viability in electrical connection strengths (Szoboszlay et al., 2016).

## Neuroplasticity of chandelier cell

Like other neurons, ChCs undergo neuroplasticity and display substantial variability in their inhibitory synapses, depending on the developmental stage and characteristics of the post-synaptic PyN activity. Here we review molecular mechanisms related to axonal development and synaptogenesis and plasticity during the development and activity-dependent variability of ChCs.

### Axo-axonic synaptic plasticity during development

The development of the ChC axon includes several key stages: Filopodia extends from axonal shafts to recognize cues and direct the axon to its final destination. After the axon arborizes, synaptic boutons develop to form the characteristic cartridges of ChCs. Finally, ChC axons selectively establish synaptic contact with the PyN AIS. Recent studies have illuminated the various cellular and molecular components responsible for these developmental processes.

The initiation of filopodia in ChCs and resulting axonal arborization is regulated by long-range cholinergic projections from the basal forebrain (BF) (Steinecke et al., 2022). When nicotinic acetylcholine receptors (nAChRs) were blocked in ChCs of the mPFC using an antagonist selective for the α4-subunit-containing isoform of the receptor, a significant decrease in filopodia growth was observed. The spiking properties of the same ChCs were not affected, indicating that the effect of acetylcholine (ACh) signaling on axons is direct and local (Steinecke et al., 2022). Moreover, the initiation of filopodia growth was observed preferentially at axonal varicosities. Therefore, cholinergic modulation is critical for filopodia formation at axonal varicosities of ChCs (Steinecke et al., 2022). Furthermore, using electroporation and calcium imaging, it was shown that T-type voltage-gated calcium channels (VDCCs) maintain the basal calcium level range in axonal varicosities. Calcium levels were reduced in varicosities when nAChRs were blocked and increased when nicotine was rapidly administered, and an increase in calcium level was followed by filopodia initiation. As a result, the α4-nAChR–T-type VDCC signaling axis regulates filopodia initiation in ChCs (Steinecke et al., 2022). The *in vivo* function of the nAChR–T-type VDCC signaling pathway in ChC axonal arborization was tested using a loss-of-function experiment. When ChCs with mutant α4-nAChRs or mutant T-type VDCCs were transplanted in developing mice, a significant decrease in axonal branching was observed at P13 compared to the wild type, confirming that the nAChR–T-type VDCC signaling axis regulates the arborization of ChC axons.

The morphogenesis of ChC synaptic boutons has been shown to be mediated by three molecules: ErbB4, DOCK7, and FGF13. First, the depletion of a receptor tyrosine kinase ErbB4, which is expressed by PV-positive interneurons, led to a decrease in ChC bouton density without affecting the overall morphology of the ChC (Fazzari et al., 2010). Second, a guanine nucleotide exchange factor DOCK7 was shown to act as a cytoplasmic activator of ErbB4 and promote ChC bouton development by augmenting ErbB4 activation independently of its GEF activity. Indeed, defective ErbB4/DOCK7 signaling was correlated with a decrease in both size and density of ChC boutons (Tai et al., 2014). Therefore, the development of ChC boutons is understood to be controlled by DOCK7's modulation of ErbB4 activity. A recent study using RNA sequencing and whole transcriptome analyses of GABAergic interneurons during early synaptogenesis found that a non-secretory growth factor FGF13 has a critical role in regulating ChC bouton development. When FGF13 was knocked out during P2 and P14, when axonal development was mostly complete, there was a significant decrease in the density of pre-synaptic ChC boutons (Favuzzi et al., 2019). The molecules ErbB4, DOCK7, and FGF13 are involved in the development of ChC boutons.

Given the highly precise subcellular targeting of ChC axons on the AIS of PyNs in the adult brain, it is important to understand how such synaptic target preference is established during post-natal development. During development, ChCs form both AIS and off-target varicosities that undergo distinct developmental regulation and develop an excess of off-target axonal varicosities in addition to AIS-targeting varicosities (Steinecke et al., 2017). Unlike off-target varicosities, AIS-target varicosities that predominantly contain pre-synaptic markers VGAT specifically formed synapses at AIS and persisted in young adulthood (P28) whereas off-target varicosities that lack pre-synaptic markers did not form synapses and its number decreased in young adulthood. A recent study also reported that coordinated axo-axonic innervation of particular AIS *via* en passant synapses was observed already at P14 before ChC cartridges are established (Gour et al., 2021). These suggest that subcellular synapse specificity of ChCs is predetermined and such predetermined target choice possibly corresponds to the gradual removal of off-target synapses over post-natal development. It is possible that molecular cues localized at AIS provide target recognition and synapse formation by ChCs.

*In vivo* RNA screening revealed that the selective innervation of the PyN AIS by ChC is regulated by a pan-axonally expressed L1 family member cell adhesion molecule L1CAM. When L1CAM was depleted in the embryonic neocortex, the number of PyN AISs innervated by ChCs decreased significantly at P28 (Tai et al., 2019). Furthermore, the number of vesicular GABA transports (VGAT) and gephyrin puncta, which are, respectively, pre- and post-synaptic markers for GABAergic synapses (Micheva et al., 2010), was also decreased at the AIS but not somatic or dendritic regions of the PyN (Tai et al., 2019). In addition to being critical for establishing ChC/PyN AIS innervation, L1CAM was shown also to play an important role in maintaining these synapses during adulthood. When most axo-axonic synapses are established in adulthood, the silencing of L1CAM in PyNs led to a significant decrease in the number of ChC/PyN synapses and gephyrin puncta per PyN AIS (Tai et al., 2019). L1CAM alone does not sufficiently explain the subcellular specificity of the ChC/PyN AIS innervation, since L1CAM is distributed pan-axonally along the PyN. A cytoskeletal complex of ankyrinG (AnkG) and βIV spectrin at the PyN AIS has been suggested as a model to anchor and cluster L1CAM molecules to promote high-affinity cell adhesion to nearby ChC cartridges (Tai et al., 2019). Supporting this model, disruption of the L1CAM-AnkG-βIV-spectrin complex was shown to reduce the density of L1CAM distribution at the AIS and importantly impair the innervation of AIS by ChC cartridges (Tai et al., 2019). It would be of great interest to identify such molecular cues that determine the connectomic target preference of ChC axons for future study.

**Figure 2:**
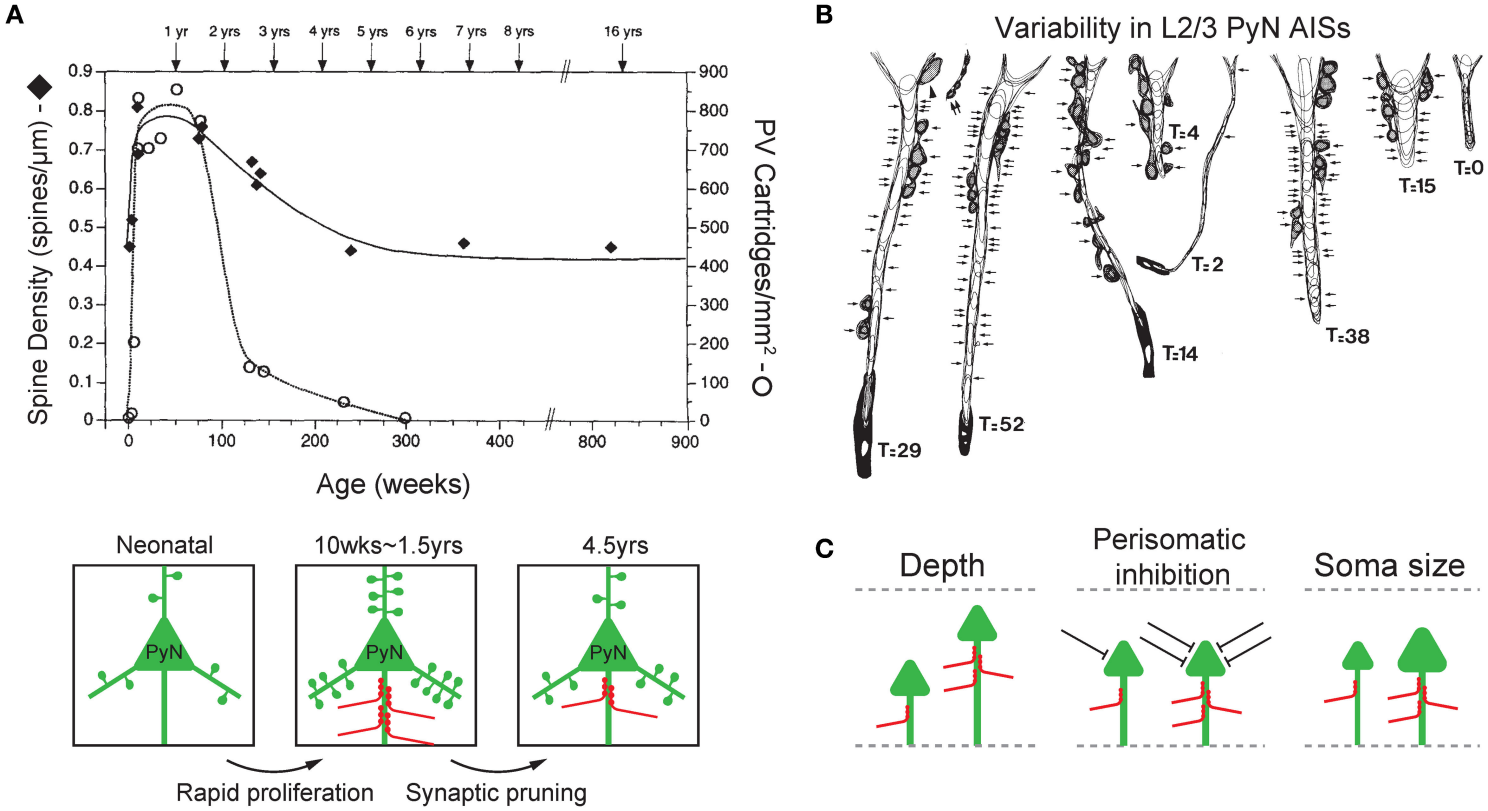
Plasticity and variability of axo-axonic synapses. **(A)** Synchronous developmental time course of changes in PyN spine density (filled diamonds) and ChC cartridge density (blank circle) in the L3 monkey prefrontal cortex. Both PyN dendritic spine density and ChC cartridge density rapidly increase during 2–3 post-natal months (rapid proliferation), reach their highest levels at 1.5 years of age, progressively decline until 4.5 years of age (synaptic pruning), then remain stable in early adulthood. Adapted from Anderson et al. (1995). **(B)** Variability in the ChC axon terminals on AISs of L2/3 PyNs of the monkey sensory-motor cortex. Arrows indicate synaptic contacts only on the AIS. *T* indicates the total number of synapses on the AIS (GAD-positive). Adapted from DeFelipe et al. (1985). **(C)** Features of target PyNs correlated with axo-axonic synaptic strength. The cortical depth of PyN soma location (left), the degree of perisomatic inhibition (middle), and the size of PyN soma (i.e. soma area and AIS radius, right) are positively correlated with the strength of ChC inputs to the AIS. Adapted from Schneider-Mizell et al. (2021).

The post-natal development of ChC axons involves an initial stage of the rapid proliferation of new synapses followed by a later stage of removal and refinement of these synapses (Pan-Vazquez et al., 2020; Gour et al., 2021; Figure 2A). In various brain regions of kittens, ChC axons branched more profusely and axon terminals displayed more complex structures when compared to those of adult cats (Somogyi et al., 1982). Specifically in the kitten visual cortex, the rapid proliferation of ChC axons and axo-axonic synaptogenesis was shown to occur up to the age of 7–8 weeks, after which the axon terminations and synaptic boutons became simplified, more prominent, and organized (Somogyi et al., 1982). *In vivo* imaging of ChCs during post-natal development showed that ChC axons rapidly arborized and formed axo-axonic synapses between P12 and P18 (Pan-Vazquez et al., 2020). In that study, they further showed that the plasticity of axo-axonic synapses is reversible and follow homeostatic plasticity rules based on developmental switches in GABAergic polarity of axo-axonic synapses from depolarizing during P12–P18 and to hyperpolarizing in older mice (P40–46). A recent study using three-dimensional electron microscopy revealed that ChC axons exhibit axo-axonic target preference for innervation of the AISs of layer 2/3 PyNs (~60% of ChC axon terminals made contact with the AIS of PyNs) by P14 and develop their full target preference with almost ~90% contact with the AIS of PyNs by P28 (Gour et al., 2021). These studies suggest that ChCs undergo significant plasticity during post-natal development, guided by various molecular and neurophysiological factors (Anderson et al., 1995; Fazzari et al., 2010; Favuzzi et al., 2019), to establish powerful inhibition on the excitatory activity of PyNs.

Interestingly, the time-course of changes in the synaptic density of inhibitory ChC synapses on the PyN AIS parallels that of excitatory synapses by other neurons on PyN dendritic spines (Figure 2A). In the monkey L3 PFC, the dendritic spines of PyNs where excitatory input is received displayed a pattern of rapid proliferation and subsequent simplification during post-natal development. PyN spine density increased rapidly during the first 2–3 post-natal months, remained high until 1.5 years of age, and progressively declined until 4.5 years of age, at which point the spine density stabilized into adulthood (Anderson et al., 1995). The same time-course of development was observed with the synaptic boutons of ChCs in the L3 prefrontal cortex that contact the AIS of PyNs. Using immunohistochemistry of PV which is found in axon terminals of ChCs, ChC boutons were first observed on 22 post-natal days, and their density substantially increased during the first 3 post-natal months. Much like the spine density of PyNs, ChC bouton density remained at its peak (of 750/mm^2^) through 1.5 years of age, then declined over the next year (Anderson et al., 1995). This close temporal parallel between the post-natal developmental pattern of PyN dendritic spines and ChC synaptic boutons is not trivial, given that the time-course of synaptogenesis greatly varies by cell type (DeFelipe et al., 1985; Schneider-Mizell et al., 2021). As such, this suggests that the plasticity of ChC axon terminals may be dependent on the level of excitatory activity of PyNs, which may function to maintain a precise balance between the inhibition exerted by ChC axo-axonic synapses and excitation by dendritic spine synapses of PyNs.

### Variability in axo-axonic synaptic strengths

The plasticity of ChC axon terminals and axo-axonic synapses is further demonstrated by the observation that there exists substantial variability in the magnitude of ChC input to PyN AISs (DeFelipe et al., 1985; Schneider-Mizell et al., 2021; Figure 2B). This variability reflects the ability of ChCs to regulate their inhibitory strength based on the characteristics of its target cell, and may have a role in shaping the functional properties of PyNs. Variability in ChC input seems to be dependent on three aspects of the innervated PyNs: laminar depth of the soma, other sources of perisomatic inhibition, and size of soma and AIS (Figure 2C).

Firstly, the laminar depth of the PyN's location is associated with ChC input strength (Figure 2C; left). Deeper PyNs receive weaker ChC inhibition. This correlation has been demonstrated in the monkey sensory-motor cortex (DeFelipe et al., 1985) and the mouse visual cortex (Schneider-Mizell et al., 2021). First, ChC axon terminals in the monkey sensory-motor cortex area 4 were stained with immunohistochemistry for the enzyme GAD which is found in GABAergic axon terminals. Doing so revealed that the AIS of PyNs in cortical layers 2 and 3 were innervated by a greater number of GAD-positive ChC axon terminals when compared to those of L5 (DeFelipe et al., 1985). In a later study of the mouse visual cortex, it was observed that even within cortical layers 2/3, greater cortical depth was associated with less number of ChC axo-axonic synapses onto a single PyN AIS hence a weaker inhibition (Schneider-Mizell et al., 2021). Second, the overall level of perisomatic inhibition, excluding ChC axo-axonic synapses, on PyN was correlated with a greater number of ChC synapses (Schneider-Mizell et al., 2021; Figure 2C; middle). Perisomatic inhibition included inhibitory synapses on the soma and non-ChC AIS synapses. Thirdly, the larger size of PyN, specifically the soma area and the radius of AIS, was associated with greater ChC input (Schneider-Mizell et al., 2021; Figure 2C; right).

That ChC axon terminals undergo significant plasticity during post-natal development and display variability in their synaptic connections to PyN AISs in adult animals supports that there is substantial variance in the strengths of the axo-axonal synapses that connect ChCs to PyNs. This variance in synaptic strength may be functionally relevant to the maintenance and regulation of the activity of PyN populations. Indeed, given the likely role of ChCs in maintaining the E/I balance of PyN populations, ChC axon terminals may demonstrate plasticity in response to varying levels of PyN population activity. Increased PyN excitatory activity may trigger the strengthening of axo-axonal synapses hence increasing levels of inhibition, to restore E/I balance and ultimately preserve the precision of the population output of PyNs. The failure to perform this function may be the underlying basis of neuropsychiatric disorders like schizophrenia, namely thought disorder, in which ChC dysfunction has been implicated.

## Chandelier cell functions

### Electrophysiological properties of ChCs

ChCs are commonly classified as fast-spiking interneurons by their electrophysiological firing (Veres et al., 2014). Upper L2/3 ChCs of the prefrontal cortex exhibit high-frequency non-adapting firing pattern and a low levels of excitability (Kawaguchi, 1995; Zaitsev et al., 2009) with moderate accommodation (Veres et al., 2014). Paired cell recordings between ChCs and pyramidal neurons in monkey DLPFC revealed that cortical ChCs have a high release probability (González-Burgos et al., 2005). Repetitive stimulation of ChCs produced frequency-dependent depression and the failure rate of ChCs was almost zero. These features indicate that inhibitory inputs from ChCs to PyNs serve as a low-pass filter providing efficient inhibition at the beginning part of the burst. Although ChCs are generally considered fast-spiking, it is noteworthy that electrophysiological properties of ChCs vary between brain regions and different species.

ChCs in different brain regions exhibit electrophysiological heterogeneity with regard to specific membrane properties. For instance, in the neocortex (Povysheva et al., 2013) and hippocampus (Buhl et al., 1994), where the majority of ChC electrophysiological recordings have been done, membranes of ChCs display different input resistance, with neocortical ChCs having a significantly higher input resistance (~167 MΩ) than hippocampal ChCs (~73.9 MΩ). Apart from this difference, neocortical and hippocampal ChCs share similar time constants of ~8 and ~7.7 ms, similar resting membrane potentials of −65 and −65.1 mV, and similar amplitude of action potentials of ~60 and ~64.1 mV, respectively. The greater input resistance of neocortical ChCs indicates either a larger axonal diameter or less number of open membrane channels compared to hippocampal ChCs. These electrophysiological differences, in addition to specific morphological differences such as the more elaborate axonal branching in the hippocampus (Li et al., 1992), may reflect a functional difference in the ways that ChCs of different brain regions uniquely contribute to the neural coding and circuitry of their respective areas.

Although fast-spiking interneurons characterized by their short, fast bursts of action potentials without adaptation do not constitute a homogeneous group, the majority of fast-spiking interneurons express the high-affinity calcium binding protein, parvalbumin (PV) and consist of two morphologically distinct cell types by the horizontal spread of the axonal arborization: basket-cells (BCs) and ChCs (Kawaguchi and Kubota, 1997; Zaitsev et al., 2005). Despite salient differences in morphology between ChCs and BCs, basic electrophysiological properties such as rapid time course, the small amplitude at resting membrane potential, and GABA-A receptor-mediated inhibition do not differ significantly between ChCs and BCs (Gonzalez-Burgos et al., 2005; Povysheva et al., 2013). For example, the functional properties of single IPSPs were qualitatively and quantitatively similar between ChCs and BCs in the monkey prefrontal cortex (Gonzalez-Burgos et al., 2005). However, in the prefrontal cortex, some differences between ChCs and BCs (firing frequency, fast and medium afterhyperpolarization, and depolarizing sag) exist across species and a difference in the first spike latency is species-specific (Woodruff et al., 2009; Povysheva et al., 2013). For example, the firing frequency of ChCs is substantially higher than of BCs in monkeys, rats, and ferrets (Krimer and Goldman-Rakic, 2001; Povysheva et al., 2013). In mouse hippocampus CA3, BCs and ChCs showed different properties (Papp et al., 2013): BCs had a lower threshold for action potential (AP) generation and lower input resistance, narrower AP and afterhyperpolarization than ChCs. BCs fire more frequently than ChCs. Thus, the differences in firing properties between ChCs and PV-BC could result from their morphological differences (Papp et al., 2013) and brain regions and in turn differently contribute to post-synaptic activity during rhythmic network oscillations in a cell-type and brain-region specific manner (Klausberger et al., 2003; Dugladze et al., 2012; Massi et al., 2012).

### Excitation and inhibition by ChCs

The axon terminals of ChCs express glutamic acid decarboxylase (GAD) which is responsible for the synthesis of GABA (DeFelipe et al., 1985), and GABA transporter (GAT)-1 which mediates GABA clearance from the synaptic cleft (Inda et al., 2007), indicating the GABAergic nature of ChCs. GABA release from ChCs activates GABA-A receptors at the post-synaptic site in PyNs (Nusser et al., 1996; Gonzalez-Burgos and Lewis, 2008) which results in the opening of chloride ion channels. Since the opening of chlorine channels typically results in the influx of chloride anions across the membrane which hyperpolarizes the post-synaptic membrane (Kaila, 1994), the effect of ChC GABAergic signaling was generally considered as inhibition.

However, several *in vitro* studies have reported that the release of GABA from cortical ChCs evokes excitatory post-synaptic responses (Szabadics et al., 2006; Molnár et al., 2008; Woodruff et al., 2009). Notably, Szabadics et al. (2006) found that PyN AISs were absent of the potassium-chloride cotransporter 2 (KCC2), which regulates the intracellular chloride concentration at the post-synaptic surface by extruding the anion (Payne et al., 1996). The absence of KCC2 at the AIS was proposed as a potential explanation for this phenomenon. The absence of KCC2 at the AIS would reduce the extrusion of intracellular chloride, leading to a reversal of the transmembrane chloride gradient and a depolarized GABA-A reversal potential (Woodruff et al., 2010). The depolarized GABA-A reversal potential would allow GABA-A receptors to mediate depolarizing events upon activation. It is noteworthy that the causal relationship between the lack of KCC2 on the PyN AIS and the depolarizing effect of ChCs has not been directly shown. To add a layer of complexity to GABAergic signaling at the AIS, an *in vitro study* suggested the role of intracellular bicarbonate (${\text{HCO}}_{3}^{-}$) levels in ensuring the inhibitory effect of the GABAergic input to the AIS of PyN (Jones et al., 2014). The level of intracellular bicarbonate (${\text{HCO}}_{3}^{-}$) regulates action potential generation in both soma and AIS *via* Kv7/KCNQ channel modulation: local ${\text{HCO}}_{3}^{-}$ efflux through GABA-A receptors at the AIS of PyN facilitates local KCNQ channel activity, which in turn greatly reduces action potential probability despite a local depolarizing Cl^−^ gradient.

In the neocortex, an *ex vivo* study showed that ChCs have depolarizing effects on PyNs under resting membrane potential yet hyperpolarizing effects under fluctuating membrane potential dynamics, suggesting the possibility of a state-dependent, dual function of ChCs (Woodruff et al., 2011). However, in the BLA, ChCs were shown to hyperpolarize PyNs even under resting membrane potential (Veres et al., 2014), similar to other *in vitro* studies in the hippocampus that demonstrated their inhibitory function (Buhl et al., 1994; Glickfeld et al., 2009). Therefore, future studies are needed to determine whether the state-dependent depolarizing effects of ChCs occur in a region-specific manner.

Studies suggesting the excitatory function of ChCs have been conducted in *in vitro* conditions. In *in vivo* studies, evidence for the inhibitory function of ChCs has been prevalent. For example, L2 ChCs in the prelimbic cortex of free-behaving mice have been shown to inhibit the firing of PyNs (Lu et al., 2017). In this study, ChCs of Nkx2.1-CreER mice were virally expressed with channelrhodopsin-2 (ChR2), and the response of target PyNs was observed using single-unit optrode recording. The optogenetic activation of prelimbic L2 ChCs monosynaptically inhibited the firing of a large number of PyNs. Similar results were reported in the CA1 hippocampus of Unc5b-CreER mice using *in vivo* extracellular recording and calcium imaging methods (Dudok et al., 2021). First, CA1 ChCs were virally expressed with ChR2, and silicone probes were used to record the activity of CA1 units in head-fixed mice during spontaneous running and resting. Optogenetic activation of ChCs resulted in rapid reductions in PyN firing rate, suggesting the inhibitory effect of ChCs. Second, ChCs were expressed with the excitatory opsin ChRmine or inhibitory opsin eNpHR and the entire CA1 neuronal population with GCaMP6f for two-photon calcium imaging. Activation of ChCs through ChRmine reduced the number of transients in PyNs, while inhibition through eNpHR significantly increased transient rates. Therefore, these results collectively suggest that the *in vivo* function of ChCs in adult animals is inhibitory.

Despite the increasing number of *in vivo* studies that have provided an understanding of the function of ChCs in live animals, the question of whether ChC synapses are depolarizing or hyperpolarizing is still not fully understood regarding different post-natal developmental periods and brain states. Developmental considerations may explain the observations of depolarizing effects of GABAergic release by ChCs reported in previous *in vitro* studies (Szabadics et al., 2006; Molnár et al., 2008; Woodruff et al., 2009). GABA is generally thought to be excitatory only during early development until around P7, when the efflux of chloride ions due to an intracellular regulation causes the membrane potential to rise above the threshold (Owens and Kriegstein, 2002; Wang et al., 2016). A recent study showed that GABAergic signaling at the AIS of mouse prefrontal PyNs switches polarity from depolarizing to hyperpolarizing over a protracted periadolescent period based on developmentally changed functions of KCC2 and NKCC1 (sodium potassium chloride cotransporter 1) (Rinetti-Vargas et al., 2017), suggesting changing role of ChCs over post-natal development.

### Neuromodulation of ChCs

Cortical brain states can be effectively regulated by neuromodulators such as noradrenaline (NA), acetylcholine (ACh), dopamine, and serotonin. Such neuromodulatory control, which can modulate the activity of cortical GABAergic neurons, plays a critical role in mediating plasticity for circuit wiring and information processing (Yaeger et al., 2019; Steinecke et al., 2022). In the neocortex, NA and ACh are released from afferent axonal fibers predominantly originating from the locus coeruleus and the nucleus basalis of the basal forebrain, respectively. In the rat frontal cortex, NA or α-adrenergic agonist, 6-fluoronorepinephrine, directly affects the activities of most GABAergic cell types by inducing depolarization but not action potential firing in fast-spiking cells including multipolar cells and ChCs (Kawaguchi and Shindou, 1998). Regarding the effect of ACh in the neocortex, activation of muscarinic receptors (mAChRs) generally increases PyN firing *via* direct depolarization and/or enhances the intrinsic excitability of PyNs (Obermayer et al., 2017). Muscarinic 1 receptors are widely expressed on somatodendritic domains of L2/3 and 5 PyNs and INs, where they increase membrane excitability (Ballinger et al., 2016). In contrast, Muscarinic 2 receptors are typically expressed in the pre-synaptic domain where they inhibit ACh release on local inhibitory GABAergic terminals to decrease GABA release (Disney et al., 2006). In the mouse PFC, carbachol, an ACh receptor agonist, potentiated the excitatory synaptic currents onto PV-BCs in L3–6, but not onto PV-BCs and ChCs in the superficial layer (Tikhonova et al., 2018). ACh can regulate the function of perisomatic inhibitory cells by modulating their GABA release (Lawrence, 2008). GABA release in PV-BCs, CCK-BCs, and ChCs is depressed by cholinergic receptor activation (Fukudome et al., 2004; Szabó et al., 2010). Cholinergic receptor activation by carbachol does not significantly depolarize fast-spiking cells (Kawaguchi and Shindou, 1998). Carbachol significantly reduced the amplitude of uIPSCs in PV-BCs and ChCs, and the reduction was restored by M2-type muscarinic receptor-preferring antagonist (Szabó et al., 2010). Furthermore, carbachol changed the short-term dynamics of GABA release: it accelerated the decay of uIPSCs in ChC-PyN pairs but not in fast-spiking BC-PyN pairs. In addition, carbachol significantly suppressed or even eliminated the short-term depression of uIPSCs in fast-spiking BC-PyN and ChC-PyN pairs in a frequency-dependent manner (Szabó et al., 2010). These suggest that ACh can differentially control the impact of perisomatic GABA release from different sources. It appears that the effects of neuromodulators including dopamine and serotonin on ChCs remain elusive. Although electron microscopy revealed that cortical PV-INs receive direct synaptic inputs from dopaminergic axons (Sesack et al., 1998), it is unclear whether dopaminergic axons exhibit distinct projections to PV-BCs and ChCs. An *in vitro* study reported that a ChC in the rat sensorimotor cortex did not respond to serotonin (5-HT) (Foehring et al., 2002). To determine specific functions of cortical ChCs in neural circuits and brain function, systematic future studies on development, brain states, and neuromodulation are needed.

## Behavioral function of ChCs

The electrophysiology and connectivity of ChCs have been characterized by many *in vitro* studies, which allowed an understanding of its function of powerfully inhibiting the action potential of PyNs and regulating the population output of PyN ensembles (Buhl et al., 1994; Veres et al., 2014). With recent advancements in genetic labeling techniques, studies have begun demonstrating the *in vivo* activity of ChCs in various brain areas and its functional relevance in the behavior of live animals.

### Role of ChCs in brain oscillations

Brain rhythm indicates highly coordinated neuronal activity underlying cognitive processes. For example, sharp wave-ripple complexes (SWRs), which have been postulated to arise from a synchronous burst of PyN population, are required for memory consolidation (Csicsvari et al., 2000). Diverse features of interneurons allow synaptic inhibition of PyNs at various subcellular compartments and temporal regulation of PyN activities with unique patterns. These interactions between interneurons and networks of PyNs determine brain rhythm oscillations (Klausberger and Somogyi, 2008). Particularly, gamma oscillations (30–80 Hz) are critical for important cognitive functions such as attentional selection (Vinck et al., 2013), working memory operations (Carr et al., 2012), perception (Melloni et al., 2007), conceptual categorization (Engel et al., 2001), and hippocampal functions such as learning and memory (Colgin and Moser, 2010). Disrupted gamma oscillation is associated with cognitive deficits in schizophrenia such as the disorganization of thought (Lewis et al., 2005; Cho et al., 2006). Fast spiking PV-expressing GABAergic interneurons such as PV-BCs and ChCs have been associated with gamma oscillations since they provide strong, phasic, and synchronous inhibition to networks of PyNs *via* their innervation of perisomatic compartments (Bartos et al., 2007; Gonzalez-Burgos and Lewis, 2008; Sohal et al., 2009).

Perisomatic inhibition at gamma frequency plays an important role in determining the spiking timing of PyNs within the theta cycle (Bartos et al., 2007; Gonzalez-Burgos and Lewis, 2008; Sohal et al., 2009). Indeed, both PV-BCs and ChCs have a high discharge probability in the descending phase of the theta when the discharge probability of PyNs is lowest and gamma power is highest (Buzsáki, 2002). However, distinct firing patterns between PV-BCs and ChCs have been reported. In the rat hippocampus CA3, PV-BCs fire at high frequency and are phase-locked to sharp wave ripple oscillation while ChCs preferentially and rhythmically fire around the peak of the theta cycles and increase firing probability at the beginning of the sharp wave episode and become saline at the maximum amplitude and after the sharp wave (Klausberger et al., 2003). Recent studies reported heterogeneous dynamics of ChCs during the sharp wave ripples (Varga et al., 2014; Geiller et al., 2020). In the rat prelimbic cortex, *in vivo* extracellular recording revealed that during DOWN- to UP-state transitions of slow oscillations, when spindle oscillations occur, PV-BCs and PyNs increased their firing rate earlier than ChCs, showing differential coupling to gamma and spindle oscillations between PV-BCs and ChCs (Massi et al., 2012). These suggest different contributions of ChCs and PV-BCs to the temporal organization of PyN network activity.

Temporal coupling of ChCs to theta and spindle oscillations rather than gamma oscillation has been suggested as their contribution to the dynamic selection and control of neuronal ensembles (Massi et al., 2012; Dudok et al., 2021). While PV-BCs are widely accepted to mediate the generation of gamma oscillations (Massi et al., 2012), the role of ChCs in generating gamma oscillations is unclear (Bartos et al., 2007; Tukker et al., 2007). Indeed, the microcircuitry of PyNs and PV-BCs can generate gamma frequency oscillations without the involvement of ChCs, evidenced by an *in vitro* study suggesting that PV-BCs but not ChCs play a central role in the generation of cholinergically induced oscillations in hippocampal slices, one of the most studied *in vitro* models of gamma oscillations (Gulyas et al., 2010). Moreover, ChC activity is more strongly coupled to the theta cycle than the gamma cycle (Klausberger et al., 2003; Klausberger and Somogyi, 2008). One possible explanation for distinct contributions between PV-BCs and ChCs to brain oscillations was that the differential synaptic localization of GABA-A receptor subunits such as α1 and α2 subunits on the somata and AIS domains of post-synaptic PyNs (Nusser et al., 1996) may underlie cell-type specific association with high- or low-frequency oscillations, depending on the IPSC duration based on the kinetics of GABA-A receptor subunit composition. For instance, the kinetics of α2-subunit-containing GABA-A receptors post-synaptic to ChCs appears to be too slow to drive gamma oscillation, which requires a fast decay of the inhibitory post-synaptic current in PyNs (Gonzalez-Burgos and Lewis, 2008). However, paired recording from interneurons and PyNs in the basal nucleus of the amygdala showed that unitary inhibitory post-synaptic currents (uIPSCs) originating from PV-BCs and ChCs are similar in the magnitude of peak amplitude and the decay time constant, but different in the latency measured at unitary connections between interneurons and PyNs (Barsy et al., 2017). A recent study using the face-matched mirror replica immunogold labeling showed similar GABA-A receptor subunit composition in perisomatic synapses made by distinct interneuron types including ChCs, PV-BCs, and CCK-BCs (Kerti-Szigeti and Nusser, 2016), suggesting that ChCs and BCs are likely to have similar post-synaptic regulation. It is noteworthy that paired recordings from interneurons and PyNs revealed a longer decay time of uIPSCs in ChC-PyN pairs than in fast-spiking BC-PyN pairs (Szabó et al., 2010). Different decay kinetics might be due to the spillover of GABA between release sites, which can result from high release probability. Cholinergic receptor agonist carbachol reduced GABA release probability from the terminals without directly altering GABA receptor functions (Behrends and Bruggencate, 1993). Carbachol accelerated the decay of uIPSCs in ChC-PyN pairs but not in fast-spiking BC-PyN pairs (Szabó et al., 2010). Given similar GABA-A receptor subunit composition at the perisomatic inhibitory synapses (Kerti-Szigeti and Nusser, 2016), different decay kinetics between ChCs and BCs could be due to cholinergic modulation of synaptic inhibition and the cross-talk of neighboring synapses (Szabó et al., 2010), not solely due to distinct GABA-A receptor subunit composition. In addition, an *in vivo* study showed that ChCs significantly increased their firing during arousal which switched the brain states from slow to theta oscillations in the hippocampus and a low-amplitude desynchronized field potential in the prelimbic cortex while BCs and PyNs did not change their firing (Massi et al., 2012). Given arousal is often associated with cholinergic signaling, different modulation by cholinergic receptor activation between ChCs and BCs may result in a distinct contribution to theta oscillation in a state-dependent manner. The anatomical differences between PV-BCs and ChCs can be related to their distinct involvement of brain oscillations. PV-BCs and ChCs have different dendritic arborizations and locations of their soma. They receive spatiotemporally distinct patterns of excitatory synaptic inputs from local PyNs, long-range thalamocortical connection, and neuromodulatory inputs, and in turn form different recurrent feedback excitation and inhibition in a microcircuit (Gonzalez-Burgos and Lewis, 2008; Andrási et al., 2017). The distinct wiring features and output properties between BCs and ChCs may differentiate control of the spike-timing of PyNs (Vereczki et al., 2016). Future study is needed to clarify the functional involvement of ChCs in gamma or theta oscillations during behavior, which is important to understand the impact of ChC dysfunction in cognitive processes.

### Role of ChCs in various brain regions

**Figure 3:**
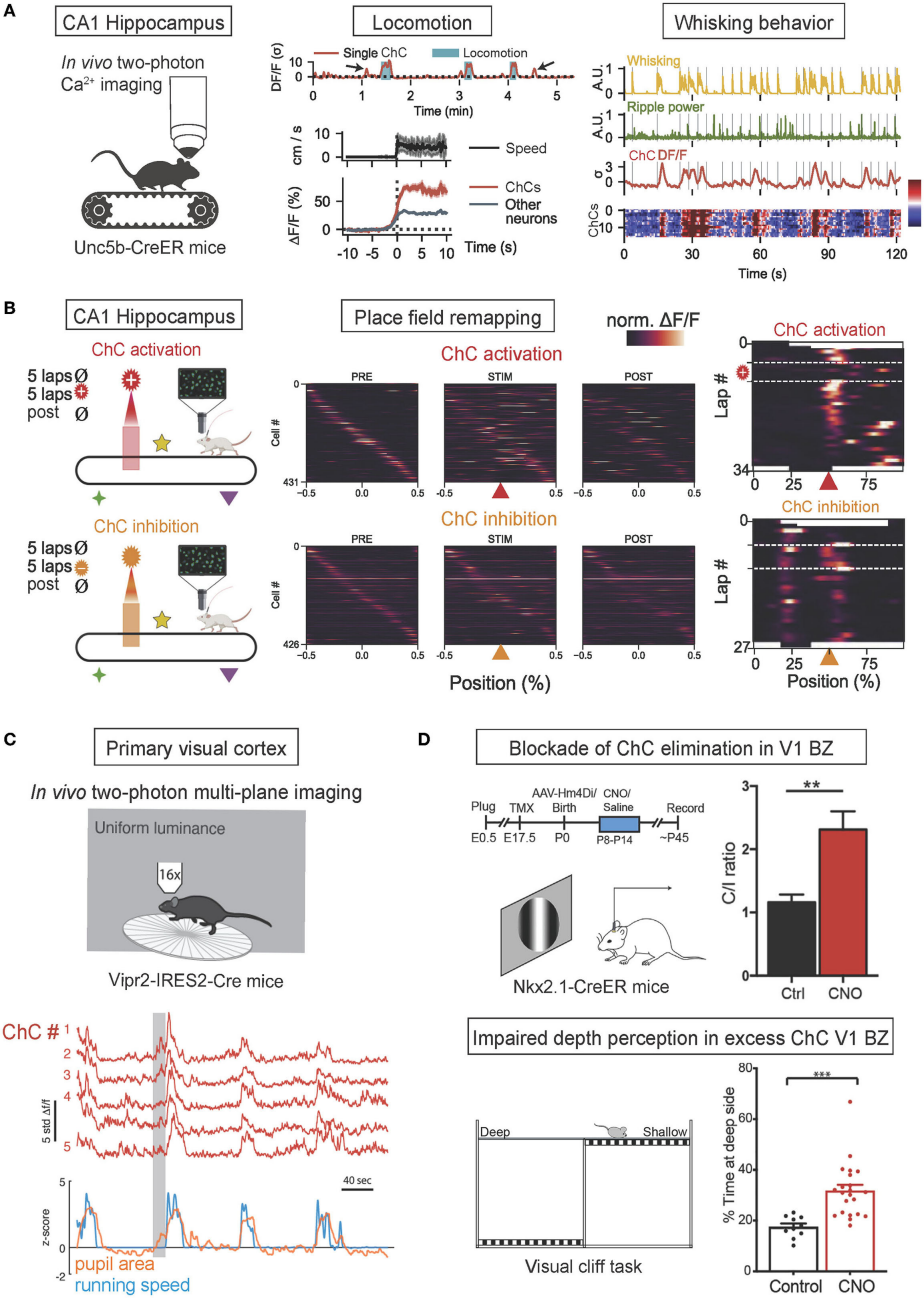
*In vivo* functions of ChCs during behavior. **(A)** *In vivo* two-photon calcium imaging of ChC activity in ChC-specific Unc5b-CreER mice on a treadmill. The increase of ChC activity in the CA1 hippocampus is associated with the onset of locomotion and whisking behavior. The concurrence of increased ChC activation (higher than other neurons) with greater running speed and higher frequency of whisking. Adapted from Dudok et al. (2021). **(B)** Simultaneous two-photon calcium imaging and spatially-locked optogenetic manipulation of CA1 ChCsl. Head-fixed Unc5b-CreER mice were allowed to run on a cue-rich treadmill (PRE), then photostimulation was applied in a fixed spatial location on the treadmill (STIM), and finally, the mice ran with no manipulation (POST). Spatial tuning curves of PyN place cells during PRE, STIM, and POST phases demonstrated that optogenetic ChC activation with ChRmine expression suppressed in-field activity of place cells (top), exhibiting the transient loss of place fields while ChC silencing with eNpHR expression induced novel place fields (bottom). Adapted from Dudok et al. (2021). **(C)** *In vivo* two-photon calcium imaging of ChC activity in ChC-specific Vipr2-IRES-Cre mice. Coordinated activity of ChCs in the V1 in freely behaving mice subjected to a uniform luminance visual stimulus was correlated with increased pupillary dilation during locomotion, a proxy for the state of arousal. Adapted from Schneider-Mizell et al. (2021). **(D)** Blockade of ChC elimination in the V1 binocular zone (BZ) results in deficient binocular vision. Timeline of experiments and neuronal responses in ChC-excess V1 (top). Clozapine-N-oxide (CNO) was applied to the V1 during P8-P14 to block ChC elimination at ~P30, resulting in excess ChC and shifting contralateral/ipsilateral responses ratio (C/I ratio) in CNO-treated mice. Schematic of visual cliff test for binocularly guided visual behavior (bottom). CNO-treated mice spend more time over at the deep side, suggesting deficits in binocular vision in excess-ChC V1 mice. **p < 0.01, ***p < 0.001. Adapted from Wang et al. (2021).

ChC activity in the hippocampus has been shown to be associated with locomotion and whisking behavior and to regulate the creation of hippocampal place fields. In a recent *in vivo* optogenetic study (Dudok et al., 2021; Figure 3A), CA1 hippocampal ChCs of Unc5b-CreER mice were selectively labeled by their expression of Unc5b (a netrin receptor highly specific to ChCs) (Paul et al., 2017) and virally expressed with GCaMP6f for two-photon calcium imaging and all CA1 neurons with jRGECO1a to monitor control activation levels. During voluntary running and resting on a treadmill, ChC activation was maximal during periods of locomotion and exceeded the activation of other CA1 neurons. Furthermore, during periods of rest, transient increases in ChC activity were observed during the onset of whisking movements, with the intensity of transients correlated to the duration of whisking. The presentation of visual or tactile sensory stimuli did not activate ChCs unless accompanied by whisking behavior. These results collectively suggest that hippocampal ChCs activate during locomotion and whisking behavior yet are not affected by sensory stimuli in the absence of movement (Dudok et al., 2021). In the same study, head-fixed mice were allowed to freely explore a cue-rich treadmill while ChCs were photostimulated at a selected location. *In vivo* optogenetic activation of ChCs resulted in the transient disappearance of hippocampal place fields at the selected location while inhibition resulted in the addition of novel place fields that persisted for long periods. Therefore, these results suggest that ChCs have a role in regulating hippocampal place fields, possibly by controlling the activity of hippocampal PyN populations which are widely known to be the neural substrates of place fields (O'Keefe and Nadel, 1978; Figure 3B).

In a recent *in vivo* study, ChC activity in the primary visual cortex (V1) has been shown to be associated with pupillary dilation and locomotion (Schneider-Mizell et al., 2021; Figure 3C). V1 ChCs of Vipr2-IRES2-Cre mice were selectively labeled by their expression of genetic marker Vipr2 and virally expressed with GCaMP6f. Head-fixed mice were exposed to a screen of uniform luminance and allowed to behave spontaneously. ChCs demonstrated seconds-long coordinated activity in which all recorded ChCs were concurrently activated, and these episodes of coordinated activity were strongly correlated with episodes of pupillary dilation during locomotion, which is known to be a proxy for arousal states (McGinley et al., 2015; Reimer et al., 2016). Therefore, these results suggest that coordinated ChC population activity is associated with naturally occurring states of high arousal marked by pupillary dilation during locomotion.

In another *in vivo* study, ChCs in the binocular zone (BZ) of V1 have been shown to play an important developmental role in binocular vision by undergoing massive apoptosis in response to retinal and callosal activity (Wang et al., 2021; Figure 3D). V1 ChCs of Nkx2.1-CreER:Ai14 mice were selectively labeled by tamoxifen administration during pregnancy. The proper developmental elimination of ChCs at the BZ through apoptosis was shown to be mediated by transcallosal inputs from the contralateral visual cortex and pre-vision retinal activity. When the proper elimination of ChCs was prevented by suppressing transcallosal inputs, BZ neurons displayed a significantly reduced responsiveness to stimulation of the ipsilateral eye, resulting in a contralateral eye-dominated V1 and deficient binocular vision, as shown by impaired depth perception. Therefore, these results suggest the crucial role of ChC elimination at the BZ in the proper development of binocular vision.

ChC activity in the basolateral amygdala (BLA) has been shown to increase in response to noxious stimuli (Bienvenu et al., 2012). In an *in vivo* study, the activity of single BLA ChCs of rats was recorded while pinches and electrical shocks were delivered to the contralateral hindpaw. In response to the stimuli, ChCs consistently and dramatically increased their firing rates with short latency, which rapidly adapted and curtailed upon stimulus offset. The BLA is known to cooperate with the hippocampus to regulate the formation of emotional memories (Maren and Fanselow, 1995; Richardson et al., 2004). Therefore, these results suggest that the activity of ChCs is involved in the process of emotional memory formation.

ChC activity in the prelimbic cortex was also shown to increase in response to noxious stimuli (Massi et al., 2012). In an *in vivo* study, the activity of prelimbic ChCs of rats was recorded while their tails were pinched. In response to the stimuli, ChCs increased their firing rates, accompanied by a switch in brain state from slow to theta oscillations. In contrast, the average firing rates of BC and PyN populations in the mPFC were unaffected by the stimuli. These results suggest the inhibitory role of ChCs in counteracting the impact of excitatory inputs from cortical and subcortical areas to allow firing only by the most excited PyNs (Massi et al., 2012).

## Role of ChCs in neural coding

By virtue of its strategic, exclusive connectivity onto the AIS of PyNs where the action potential is generated with the highest likelihood after diverse somatodendritic excitatory inputs arrived at the soma, ChCs have been generally thought to exert an effective inhibitory control on PyN outputs (Somogyi, 1977). ChCs effectively inhibit the firing of PyN or delay spike generation by 30 ms if ChC inhibition preceded PyN spiking by no more than 80 ms (Veres et al., 2014).

While PV-basket cell and PyNs connectivity are extensively reciprocal and largely non-selective, ChC-PyNs connectivity is directional and highly selective (Lu et al., 2017). In addition to the difference in connectivity to PyN populations, computational and experimental studies suggested that ChC inhibition effectively controls the threshold for action potential generations in PyNs while BC inhibition controls the suprathreshold discharge (Douglas and Martin, 1990; Veres et al., 2014). It offers a mechanism for ChCs enhancing the signal-to-noise ratio in population activity in which small signals are blocked by ChC inhibition while larger signals are relatively unaffected. Recent *in vivo* studies showed that ChCs fire in synchrony during high arousal states (Dudok et al., 2021; Schneider-Mizell et al., 2021). Given that the AIS of a single PyN receives the summed inhibitions from the afferent axons of multiple ChCs (Tamás and Szabadics, 2004; Veres et al., 2014), the synchronized ChC inputs can efficiently veto action potential generation in PyNs receiving moderate excitatory inputs, leaving out selective PyN activity receiving strong excitatory inputs. Since a single ChC can delay spike generation by 10–30 ms (Veres et al., 2014), ChCs provide the ability to regulate spike timing-dependent plasticity by controlling the precise time of PyN spiking. In addition, activity-dependent plasticity in ChC-PyN connections offers a mechanism to set a threshold of action potential generations as a function of individual neurons' excitability (Grubb and Burrone, 2010; Kuba, 2012; Wefelmeyer et al., 2015). These functions of ChCs may suggest its role in shaping neuronal outputs at the population level and selecting neuronal ensembles to route information flow dynamically.

Properties of synaptic and neuromodulatory inputs to ChCs are also important to understanding the impacts of ChCs on PyN population activity. L2 ChCs exhibit predominant apical L1 dendrites and electrical stimulation of layer 1 recruits ChC-mediated feedforward inhibition on L2/3 PyNs (Woodruff et al., 2011). Major sources of L2 ChCs include sparse local excitatory inputs and more diverse sources from local inhibitory neurons, the thalamic nuclei implicated in working memory and behavioral flexibility (Parnaudeau et al., 2013) such as the mediodorsal, anteromedial, and ventromedial thalamic nuclei, and the cholinergic inputs from the basal forebrain associated with arousal (Jiang et al., 2015; Lu et al., 2017). These inputs innervate the predominant apical layer 1 dendrites of ChCs, providing top-down, highly processed information to ChCs depending on the behavioral state of the animal (Woodruff et al., 2011). Thus, L1 dendritic integrations and feedforward inhibitory control of PyN population in ChCs can be regarded as an important computational unit for providing state-dependent top-down control on the formation and dynamics of neuronal assemblies in cortical networks.

The balance of excitatory and inhibitory inputs received by a neuron plays an important role in neural circuit homeostasis and information processing in cortical networks (Vreeswijk and Sompolinsky, 1996; Shu et al., 2003; Rubin et al., 2017), and disruption of the balance is strongly associated with pathological and dysfunctional brain states including epilepsy, autism spectrum disorder, and schizophrenia (Yizhar et al., 2011; Denève and Machens, 2016). The excitatory-inhibitory (E/I) balance is referred to as the equal average amounts of depolarizing and hyperpolarizing synaptic membrane currents (Vogels et al., 2011). If a neural network is considered globally balanced, each neuron receives large but approximately equal amounts of excitatory and inhibitory inputs that result in relatively small fluctuations in total synaptic input by canceling each other. Experimental observations suggested that excitation and inhibition are globally balanced in cortical circuits (Shu et al., 2003; Haider, 2006; Iascone et al., 2020).

**Figure 4:**
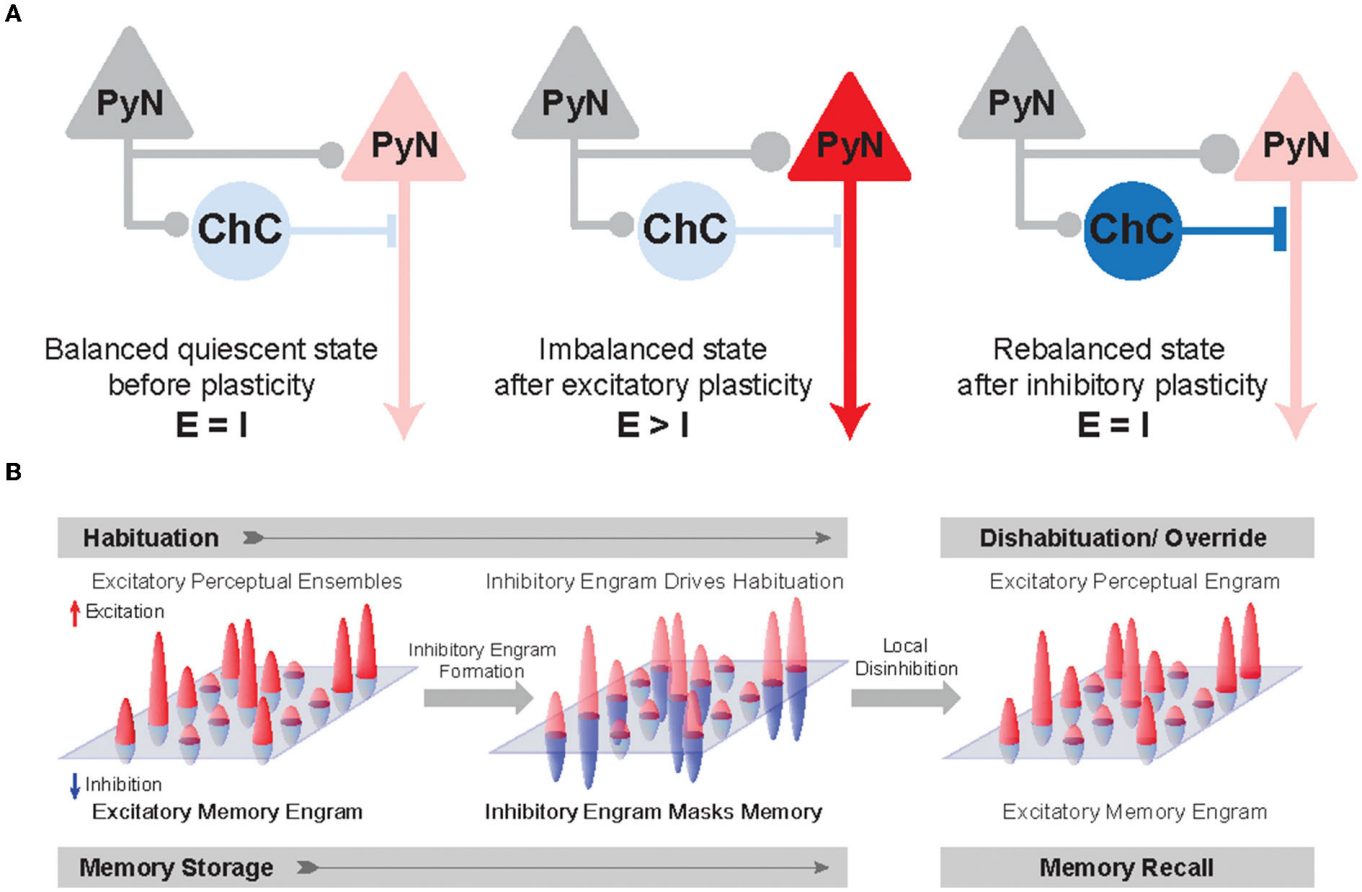
Inhibitory synapse plasticity for E/I rebalancing and inhibitory engram formation in perception and memory. **(A)** Schematic circuit diagram showing pre-synaptic PyNs (gray), post-synaptic PyNs (red), and ChCs (blue). The post-synaptic PyN receives balanced excitatory and inhibitory inputs (Balanced quiescent state). The effect of excitatory plasticity between pre- and post-synaptic PyNs initially leads to E/I imbalance (Imbalanced state). Inhibitory plasticity in axo-axonic synapses between ChCs and PyNs can subsequently restore E/I balance following excitatory plasticity (Rebalanced state). **(B)** A hypothetical framework of inhibitory engrams in perception and memory. Positive red and negative blue peaks represent excitatory and inhibitory inputs, respectively, onto an array of post-synaptic PyNs. These peaks constitute excitatory and inhibitory engrams, respectively. Regarding habituation, experiencing novel stimuli result in excitatory perceptual ensembles, but trigger relatively weak or imprecise inhibition. Repeated experiences result in the formation of a matched inhibitory engram that reduces the response and drives behavioral habituation. Regarding the memory process, memory is first encoded as excitatory engrams. Repeated experience results in the formation of matched inhibitory engrams to rebalance the array of post-synaptic PyNs. The formation of these inhibitory memory engrams may occur *via* homeostatic potentiation of inhibition onto post-synaptic PyNs that show increased levels of excitation. Inhibitory engrams allow flexible yet stable memory storage in a latent form for context-appropriate recall, which is hypothesized to occur through focused disinhibition. Adapted from Barron et al. (2017).

Given that ChCs are strategically positioned to exert powerful and selective control over outputs of PyN population (Veres et al., 2014; Blazquez-Llorca et al., 2015; Lu et al., 2017), ChCs have been suggested to ultimately contribute to keeping network excitability from going out of control by maintaining proper E/I balance. Indeed, whole-cell *in vivo* recordings revealed that ChCs, which have a low spontaneous firing rate, fire more robustly than other cortical neurons when the overall cortical excitation increases (Zhu et al., 2004). Axon terminals of ChCs are lost at the cortical epileptic foci, suggesting that disruption of ChC function contributes to the hyperexcitability of the network (Ribak, 1985). While the role of ChCs in maintaining global E/I balance serves a homeostatic function in the brain (Figure 4A), it remains elusive how ChCs contribute to dynamic information processing which is highly relevant to healthy cognition and many neuropsychiatric symptoms. Here we review a potential link between ChC and neural coding.

Percepts and memories are thought to be represented in the neuronal population by the activity of PyN ensembles often called excitatory “engrams.” The inhibitory engrams have been proposed as “negative images” or “inhibitory representation” for associative memory storage and recall (Barron et al., 2017; Figure 4B). The inhibitory engrams can be constructed in neural networks by E/I balance through homeostatic mechanisms that maintain a balance of depolarizing and hyperpolarizing currents in a neuron despite plastic changes across neurons and synapses. Plasticity of inhibitory synapses has been experimentally and theoretically proposed as a critical mechanism to create the inhibitory engrams that counterbalance new, unbalanced excitatory patterns that arise within neural networks in response to experience (Tao and Poo, 2005; Froemke et al., 2007; Vogels et al., 2011; Hennequin et al., 2017). Such experience-dependent inhibitory synaptic plasticity has been suggested to underlie precise E/I balance in time and space (Hennequin et al., 2017): the E/I balance is said to be tight if excitation and inhibitory inputs to a single neuron balance each other on fast timescales (Vogels et al., 2011; Denève and Machens, 2016) and said to be detailed if spatial patterns of excitation and inhibitory inputs to a single neuron balance each other (Vogels and Abbott, 2009). Previous experimental studies suggest that precise E/I balance provides precision and efficiency in cortical neural coding (Wehr and Zador, 2003; Isaacson and Scanziani, 2011; Zhou and Yu, 2018). Theoretical works have suggested that precise E/I balance confers the ability of neurons to gate multiple signals as a more efficient way to select for relevant features rather than suppressing all irrelevant inputs (Vogels and Abbott, 2009; Ferguson and Gao, 2018), which is consistent with the suggested role of ChC in enhancing the signal-to-noise ratio in that weak signals are blocked by AIS inhibition while strong signals pass relatively unaffected (Douglas and Martin, 1990).

Experimental evidence has suggested that ChCs play a role in controlling cell-by-cell level inhibition within a network. High variability in the number of ChC pre-synaptic inputs on the AIS of PyN has been reported in cats (Fairén and Valverde, 1980), monkeys (DeFelipe et al., 1985), and mice (Wang and Sun, 2012; Veres et al., 2014; Schneider-Mizell et al., 2021). A recent electron-microscopic study suggested that ChCs formed synapses with nearly all PyNs in L2/3 and the strength of ChC synapses adjusted for individual target cells according to cell-specific structural factors: the number of ChC synapses positively correlates with the properties of individual target cells such as the physical size of the cell and the amount of somatic inhibition (Schneider-Mizell et al., 2021), which is consistent with the notion that ChCs may provide a different degree of inhibition across individual cells to match their inputs. Furthermore, previous studies suggest that the plasticity of axo-axonic synapses at the AIS is activity-dependent (Grubb and Burrone, 2010; Kuba et al., 2010; Kuba, 2012). Presynaptic activity regulates intrinsic excitability at AIS and structural tuning of the AIS, which fine-tune neuronal excitability (Kuba et al., 2010; Kuba, 2012). In hippocampal PyNs, the increased neuronal activity causes a distal shift of the AISs, which reduces their excitability (Grubb and Burrone, 2010). In auditory neurons in birds, AISs are short and remote when synaptic inputs are strong while the AISs elongate to increase their excitability when synaptic inputs are deprived (Kuba et al., 2010; Kuba, 2012). Although activity-dependent development of GABAergic synapses has been observed in dendrites of developing brains (Oh et al., 2016; Oh and Smith, 2018), activity-dependent mechanisms for inhibitory synapse plasticity at AIS are not yet clear. However, given the strategic position of ChCs in mediating cell-to-cell level inhibition on PyN populations and activity-dependent plasticity of axo-axonic synapses, structural and functional plasticity of ChC axo-axonic synapses can be of great interest in mediating inhibitory representations and explaining key features of cognition. The potential roles of cortical ChCs in mediating detailed E/I balance may be especially important in high-level executive functions such as working memory, attentional selection, planning, and decision-making, which entail a large capacity for effective and dynamic control of signal flow in the prefrontal circuitry receiving multimodal inputs from various sensory areas, limbic areas, and neuromodulatory nuclei. Future *in vivo* experiments would be important to examine how ChCs facilitate the precision and efficiency of cortical neural codes.

## Schizophrenia and pathophysiology of ChCs

Schizophrenia is a psychiatric disorder that is associated with cognitive symptoms such as delusion, hallucinations, and disorganized thought (Elvevag and Goldberg, 2000; Telles-Correia et al., 2016). Specifically, cognitive dysfunctions in schizophrenia consist of overarching deficits in the ability to adjust thoughts or behaviors in a manner to achieve goals (Cho et al., 2006; Lesh et al., 2011). The dorsolateral prefrontal cortex (DLPFC) is the main site of aberrant electrophysiological activity reflecting neuronal network dysfunction in schizophrenia (Cho et al., 2006; Minzenberg et al., 2009). A reduction in excitation of the L3 DLPFC PyN populations has been known as a salient pathology of schizophrenia (Lewis et al., 2012), indicating a disrupted state of E/I balance. Such dysfunctions in schizophrenia are thought to be related to alterations in the inhibitory circuitry of the PFC resulting from pathological cellular changes of cortical GABAergic interneurons (Tanaka, 2008; Lewis et al., 2012; Selten et al., 2018). Recently, as emerging evidence suggests that disruption in E/I balance and interneuron dysfunction are shared for pathophysiological mechanisms of psychiatric disorders (Yizhar et al., 2011; Xu and Wong, 2018; Shaw et al., 2020), interest in the contribution of ChCs to proper circuit function in disease has been growing (Wang et al., 2016; Gallo et al., 2020). The neocortical ChC is one of the critical interneuron types that have been closely associated with schizophrenia, since cellular changes in the ChC's molecular composition, GABAergic signaling, and axon terminal structure have been rigorously documented in post-mortem schizophrenic human subjects (Pierri et al., 1999; Volk et al., 2002; Hashimoto et al., 2003, 2008; Rocco et al., 2016, 2017; Schoonover et al., 2020). Given the possibility that ChCs may be the neural substrate of precisely maintaining E/I balance required for proper information flow in the PFC, pathological alterations of ChCs may be directly linked to the cognitive symptoms displayed in schizophrenia. Here we review the current understanding of the molecular and structural alterations of ChCs in schizophrenia.

**Figure 5:**
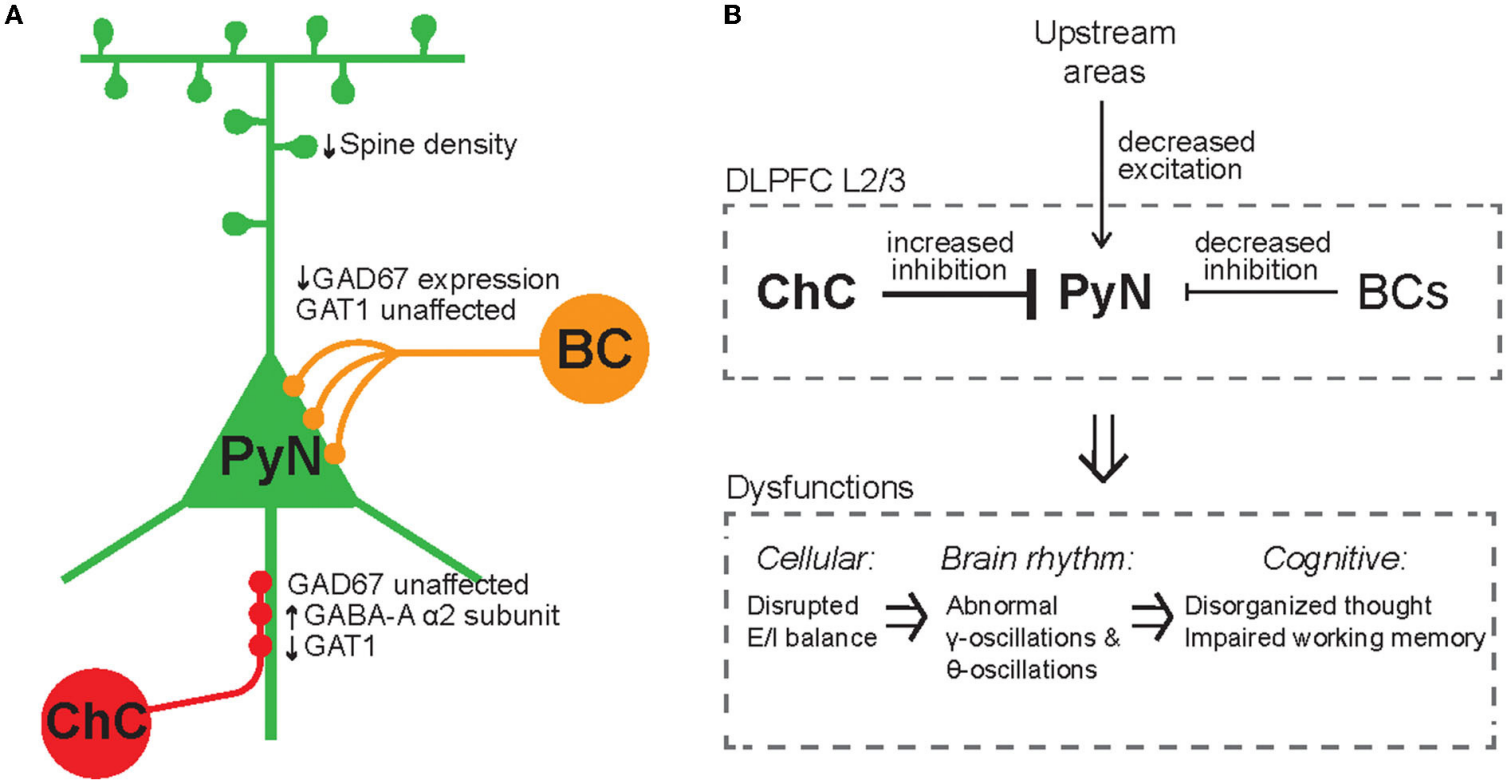
Synaptic alterations of ChCs, BCs, and PyNs in schizophrenia. **(A)** Schematic of molecular and cellular alterations of ChCs, BCs, and PyNs in schizophrenia. In ChCs, GAD67 levels in terminals are unaffected, post-synaptic GABA-A α2-subunit receptor density is increased, pre-synaptic GAT1 density is reduced, and ChC cartridge density increases. ChC-related alterations correspond to an increase in inhibition on PyNs. In BCs, GAT67 levels are decreased, suggesting a decrease of BC-mediated inhibition on PyNs. In PyNs, dendritic spine density is reduced, resulting in lower levels of excitatory drive. **(B)** GABAergic interneuron-related pathophysiology in schizophrenia. ChC-related alterations reflect an increased inhibition on PyNs, and PyNs receive lower levels of excitatory drive from upstream areas, resulting in reduced excitability of PyNs and disruption of E/I balance. BC-related alterations reflect a decrease in inhibition on PyNs.

ChCs were shown to display pre- and post-synaptic molecular alterations suggesting an increase in ChC-mediated inhibition on PyNs. First, the density of GABA transporter type 1 (GAT-1) has been reported to be significantly reduced on the axon terminal cartridges of ChCs in layers 2 to 4 in the DLPFC (Pierri et al., 1999; Volk et al., 2002; Figure 5A). Since GAT-1 is responsible for the clearance of GABA from the synaptic cleft and reduction in GAT-1 function at perisomatic synapses of DLPFC PyNs is known to prolong GABA-A receptor-mediated IPSPs (Gonzalez-Burgos et al., 2009), the effect of pre-synaptic GAT-1 density reduction in layers 2–4 is thought to be an increase in ChC-mediated inhibition on PyNs. Second, the density of GABA-A α2-subunit receptors increased on the post-synaptic AISs of PyNs in supragranular layers of DLPFC in schizophrenia, with the greatest increase shown in L2 (Volk et al., 2002; Figure 5A). Furthermore, the mRNA expression level of GABA-A α2-subunit was 14% higher in L2 of DLPFC in schizophrenia (Beneyto et al., 2011). These post-synaptic alterations of GAT-1 and GABA-A α2-subunit receptors at AISs suggest an increase in ChC-mediated inhibition on PyNs in the DLPFC in schizophrenia. It is noteworthy that these molecular alterations were not shared in other psychiatric disorders such as major depression disorder (Volk et al., 2002), indicating the distinct role of this pathophysiology in schizophrenia.

Given the general classification of ChCs as PV-INs, the specific role of ChCs in schizophrenia was obscured by observations of decreased expression of glutamic acid decarboxylase 67 (GAD67) involved in GABA synthesis in PV-INs in schizophrenia (Hashimoto et al., 2003). However, similar expression levels of GAD67 in ChCs between schizophrenia and healthy subjects (Rocco et al., 2016) differentiate their effect from the effect of GAD67 reduction shown in PV-INs. Indeed, in schizophrenia (Curley et al., 2011), PV-BC synaptic boutons in the DLPFC showed decreased levels of GAD67, suggesting that PV-BC-mediated inhibition is decreased. Thus, these results may suggest that ChCs and PV-BCs contribute to different pathophysiology of the DLPFC in schizophrenia: ChCs exert excessive inhibition on PyNs while PV-BCs may decrease inhibition to compensate for the PyN excitability. In addition, it is noteworthy that L2/3 PyNs in the DLPFC display a reduced density of dendritic spines (Lewis et al., 2012; Glausier and Lewis, 2013), reflecting a reduction in excitatory drive from upstream areas. This dendritic alteration in PyNs may also contribute to disrupted E/I balance within the DLPFC recurrent network of schizophrenia.

ChCs were shown to display structural alterations in their axonal terminals in schizophrenia. A recent study investigated the change in density of ChC cartridges in schizophrenia by taking immunopositivity to vesicular GABA transporter (vGAT) as the accurate measure for true cartridge count (Rocco et al., 2017) since neither vGAT mRNA expression level nor the level of vGAT per individual axonal bouton is affected in the DLPFC of schizophrenia (Rocco et al., 2016). It was shown that the density of ChC cartridges in schizophrenia is significantly increased in L2 of DLPFC while the number of boutons per cartridge is unaffected (Rocco et al., 2017), suggesting that ChCs may innervate a greater number of PyNs and exert greater inhibitory control over PyN populations in schizophrenia. Furthermore, the increase in ChC cartridge density in the L2 DLPFC was specific to those cartridges that expressed calbindin (CB) (Rocco et al., 2017), which is thought to be heterogeneously expressed in ChC cartridges (Rio and DeFelipe, 1997). Future studies will need to investigate the functional role of CB+ ChC cartridges in schizophrenia and the role of CB in regulating the development of ChC cartridges.

The involvement of PV-INs in schizophrenia has been suggested by numerous observations of altered gamma-band oscillations (Figure 5B), which are correlated with working memory load (Howard et al., 2003). Gamma oscillations have been shown to be lower powered in the frontal lobe of schizophrenia patients during cognitive control tasks (Cho et al., 2006). In contrast, more recent evidence has shown that, in medication-naïve, first-episode, chronic schizophrenic patients, gamma-band power is elevated in the resting state (Kikuchi et al., 2011). However, although PV-INs are thought to give rise to gamma oscillations (Bartos et al., 2007; Gonzalez-Burgos and Lewis, 2008; Sohal et al., 2009), the status of ChCs as PV-INs and the involvement of ChCs in gamma oscillations is unclear (Bartos et al., 2007; Tukker et al., 2007). Thus, abnormal gamma oscillations in schizophrenia may not reflect dysfunction of ChCs but that of PV-BCs, which comprise the majority of PV-INs.

Accumulating evidence suggests that ChCs in schizophrenia exert excessive inhibition on PyNs. The dysfunction of ChCs in schizophrenia likely has a contributory role by overly reducing the excitatory activity of PyN ensembles and in turn disrupting E/I balance (Figure 5B). Given that precise E/I balance in PyN ensembles allows for proper precise neural coding and executive cognitive function in the DLPFC, ChCs may have a far more salient role in the cognitive dysfunction of schizophrenia than previously thought.

## Summary/conclusion

Despite the diversity of GABAergic interneuron types that are thought to underlie various cortical processes and complex behaviors, the specific role of single-type interneurons remains elusive. Here we reviewed the ChC, a single-type GABAergic interneuron, in regard to the structural and functional roles of ChCs in brain circuit and neural coding and their dysfunction in neuropsychiatric conditions.

The morphology and input/output connectivity features of ChCs contribute to their functional role in neural coding. Cortical ChCs can integrate by receiving excitatory local and long-range input and cholinergic input from the basal forebrain through their apical dendrites in Layer 1 and electrically couple the activity of ChCs *via* gap junction. The iconic chandelier-like axonal arborization of ChCs exclusively innervates the AIS of neighboring PyNs, where ChCs provide efficient inhibitory control to the site of action potential generation. These structural features can provide strategic, temporally-organized inhibitory control of PyN populations based on brain states or tasks.

We reviewed the neuroplasticity of ChC axo-axonic synapses with respect to development, cholinergic modulation, and pre-synaptic molecules, which can regulate their axonal growth. A developmental pattern and a cell-adhesion molecule can regulate the target specificity of axo-axonic synapses. In addition, the parallel time course of axo-axonic synapse density with PyN excitability during development suggests the role of ChC in maintaining E/I balance in the cortical network. The variability in axo-axonic synaptic strengths positively correlates with features of target PyNs including soma location, size, and perisomatic inhibition. Given the remarkable developmental and activity-dependent plasticity of axo-axonic synapses, future studies will need to identify other molecular and neuromodulatory control mechanisms of ChC target specificity and variability of axo-axonic synaptic strengths, which are essential for the proper assembly of the cortical circuit and dynamic information processing, respectively.

ChC function depends on their electrophysiology and post-synaptic responses to their GABAergic signaling. Despite their fast-spiking property, heterogeneity in membrane properties of ChCs is found across different brain regions such as the neocortex and hippocampus, suggesting their diverse contributions to their embedded network and coding. We compared the electrophysiological features of two fast-spiking cell types, ChCs and PV-BCs, and discussed factors that may contribute to differences in their firing properties. To discern their controversial GABAergic synaptic effect, we examined *in vitro* and *in vivo* evidence of depolarizing and hyperpolarizing effects made by ChC synapses. Although several explanations have been provided for the excitatory effects of ChCs *in vitro*, emerging *in vivo* studies with ChC-specific manipulations have revealed their inhibitory effect in free-behaving adult animals. However, developmental factors and neuromodulation-dependent brain states need to be considered to understand the specific role of ChC synaptic effects. Therefore, systematic future *in vivo* studies using ChC-specific genetic markers across different developmental stages and brain states would clarify the functional features of ChCs in the post-synaptic PyN activity.

As brain rhythms indicate highly coordinated neuronal activity underlying cognitive states and behavior, we compared distinct temporal coupling of ChCs and BCs to gamma and theta oscillations. Factors such as GABA-A receptor subunit composition, state-dependent cholinergic modulation, and distinct wiring features may account for their differential contributions to gamma and theta oscillations and functional implications to PyN network activity. Recent *in vivo* studies of genetically targeting ChCs in various brain regions revealed that the activity of ChCs represents arousal states and displays strong responsiveness to salient stimuli. ChC-specific manipulation showed its direct inhibitory influence on behavioral functions of target neurons. ChC activity may actively process salient information to selectively recruit the most relevant PyN ensembles, which in turn facilitate the corresponding behaviors. The previously enigmatic behavioral functions of ChCs have been gradually unmasked through recent advancements in ChC-specific genetic labeling, optogenetics, and *in vivo* recording techniques. Yet, the active role of ChCs during cognitive tasks has not been demonstrated in the PFC where cognitive deficits are seen in schizophrenia. Future studies will need to investigate the higher-level cognitive functions of ChCs.

We discussed how ChCs shape neuronal outputs at the population level and select neuronal ensembles to route information flow dynamically. Directional and cooperative ChC-PyNs connectivity allows ChC to control the threshold for generating PyN action potentials and regulating the temporal precision of PyN spiking. This enhances the signal-to-noise ratio in PyN population codes and provides the ability to limit temporal windows for spike timing-dependent plasticity, which is necessary for shaping neural codes. Activity-dependent plasticity in ChC-PyN connections offers a mechanism to set a threshold of action potential generations as a function of individual neurons' excitability. Given the importance of E/I balance for neural circuit homeostasis and information processing, theoretical perspectives of experience-dependent plasticity of inhibitory synapses for precise E/I balance will be useful to understand how ChCs may gate multiple signals and facilitate associative memory processes through inhibitory engrams. As an underlying cellular mechanism, we discussed the activity-dependent plasticity of axo-axonic synapses that enables fine-tuned inhibition to match excitability. Thus, cell-to-cell level ChC-mediated inhibition and their activity-dependent plasticity may offer a mechanism for constructing behaviorally relevant inhibitory representations. Future *in vivo* experiments would be important to examine how cortical ChCs facilitate effective and dynamic control of information flow with precision for high-level executive functions during health and disease.

We reviewed the pathophysiological changes of ChCs in schizophrenia. Both molecular and structural alterations of ChCs in schizophrenia exert excessive inhibition on DLPFC PyNs, which may underlie cognitive deficits of schizophrenia such as disorganized thought. PV-BCs in the DLPFC appear to undergo cellular changes in schizophrenia that result in the opposite pathophysiology to ChCs: decreased inhibition of PyNs. ChC-related changes in schizophrenia seem to display laminar-specificity to the superficial layers. Although gamma oscillations have been shown to be altered in schizophrenia, the specific contribution of ChCs to gamma oscillations is unclear. Future studies will need to investigate the differential roles of laminar-specific ChCs and their involvement in altered brain activity.

In conclusion, significant progress in the research of GABAergic interneuron transcriptomic profiles, developmental biology, and functions has allowed us to recognize the association between cell-type specific dysfunction and neural disorders. While emerging evidence suggests that pathophysiological mechanisms of psychiatric disorders include disrupted E/I balance and interneuron dysfunction as shared features, we cannot fully understand diverse dysfunctional cognition or behavior without understanding the specific role of each interneuron type in neural coding. Recent *in vivo* studies with genetic targeting of ChCs have provided insights into the distinctive roles of single-type interneurons in neural computation, dissecting the complex functions of GABAergic interneurons. Future research needs to address how neocortical ChCs adaptively orchestrate dynamic PyN activity *via* axo-axonic synaptic plasticity for information processing during cognitive tasks, and what genetic or molecular factors cause defects in the development and functions of axo-axonic synapses in disease. Such efforts are crucial to specify pathophysiology and design effective therapeutic approaches. Strategy and knowledge gained from ChC studies may be utilized as a benchmark to unveil specific contributions of diverse GABAergic interneurons to circuit wiring and neural coding in health and disease at single cell-type precision.

## Data availability statement

The original contributions presented in the study are included in the article/supplementary material, further inquiries can be directed to the corresponding author.

## Author contributions

KJ and YC did literature search and wrote the manuscript draft. KJ, YC, and H-BK edited the manuscript. All authors contributed to the article and approved the submitted version.

## Funding

This work was supported by Johns Hopkins School of Medicine (to H-BK) and DP1MH119428 (to H-BK).

## Conflict of interest

The authors declare that the research was conducted in the absence of any commercial or financial relationships that could be construed as a potential conflict of interest.

## Publisher's note

All claims expressed in this article are solely those of the authors and do not necessarily represent those of their affiliated organizations, or those of the publisher, the editors and the reviewers. Any product that may be evaluated in this article, or claim that may be made by its manufacturer, is not guaranteed or endorsed by the publisher.
